# ROS-generating nanoplatforms as selective and tunable therapeutic weapons against cancer

**DOI:** 10.1186/s11671-023-03939-w

**Published:** 2023-12-11

**Authors:** Federica Foglietta, Loredana Serpe, Roberto Canaparo

**Affiliations:** Department of Drug Science and Technology, University of Torino, Via Pietro Giuria 13, 10125 Turin, Italy

**Keywords:** Reactive oxygen species, Nanomedicine, Cytotoxic therapy, Cancer models

## Abstract

Reactive species refers to a group of chemicals, mainly reactive oxygen species (ROS) and reactive nitrogen species (RNS), that are naturally formed by cells as a byproduct of cell metabolism and regulated by various internal and external factors. Due to their highly chemical reactivity, ROS play a crucial role in physiological and pathological processes which is why studies on ROS regulation for disease treatment show attracted increasing interest. Notably, ROS are now studied as a powerful therapeutic weapon in ROS-regulating therapies such as ROS-based cytotoxic therapies mediated by ROS-increasing agents for cancer treatment. Thanks to the significant progress in nanotechnology, innovative nanoplatforms with ROS-regulating activities have been developed to look for effective ROS-related nanomedicines. In this review, studies on ROS-based cytotoxic therapies against cancer as photodynamic therapy (PDT), sonodynamic therapy (SDT), radiation therapy (RT) and chemodynamic therapy (CDT) are discussed, with a focus on the stimuli-responsive ROS-generating nanoplatforms developed for breaking the current therapeutic limits of ROS-based cytotoxic therapies. Finally, we suppose that our review on this developing field will be valuable for promoting the progress of ROS-based cytotoxic therapies not only in basic research but overall, in translational research and clinical application.

## Introduction

**Fig. 1:**
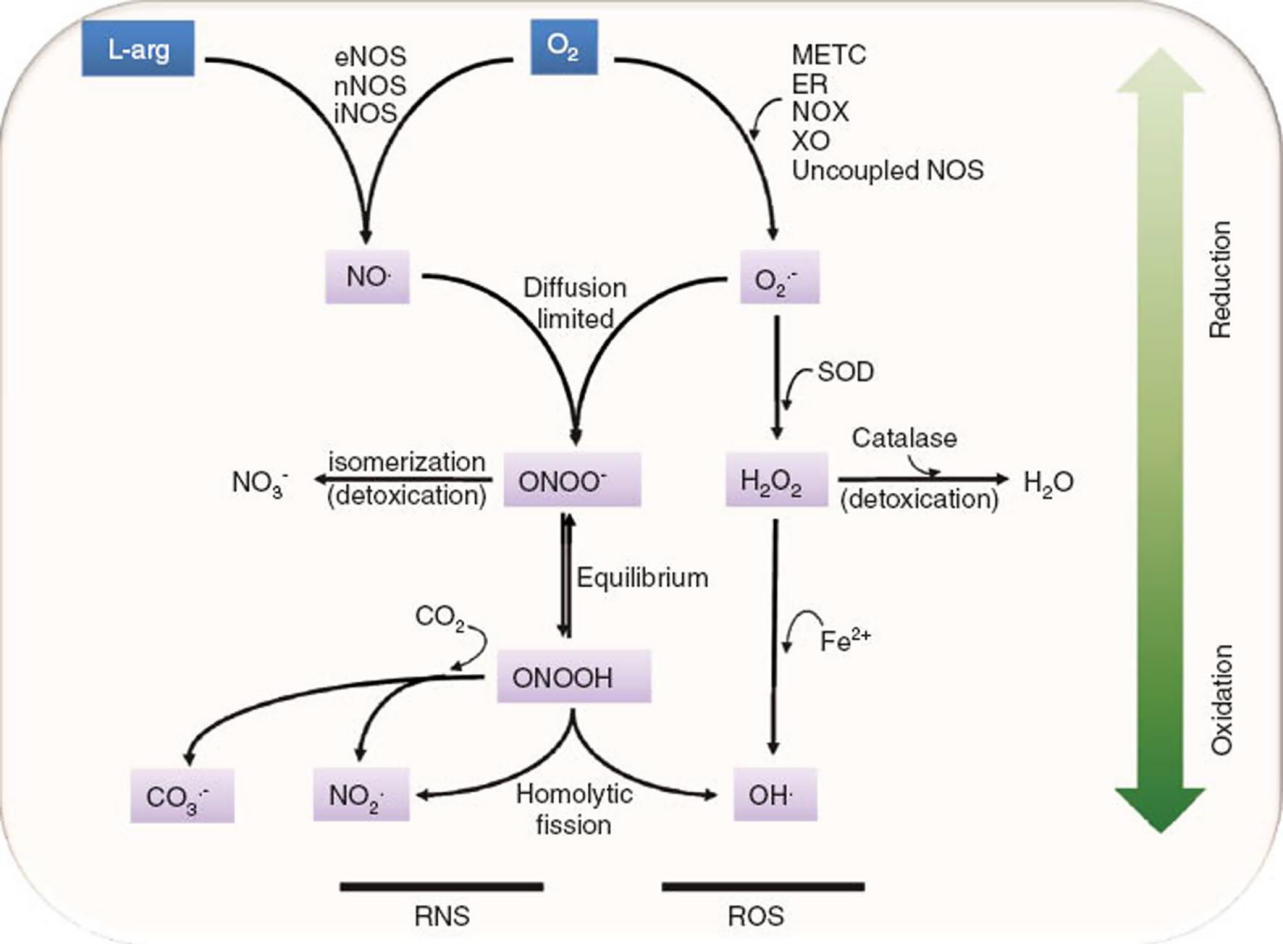
Molecular pathways of RNS and ROS generation. Nitric oxide (NO^•^) is formed from l-arginine and molecular oxygen (O_2_) by the activity of various isoforms of NO synthase (NOS) (endothelial (e), neuronal (n), and inducible (i) NOS). The superoxide radical (O_2_^•−^) is formed during cellular metabolism in the mitochondrial electron transport chain (METC) and in the endoplasmic reticulum, or as a product of the enzymatic activities of NADPH oxidase (NOX), xanthine oxidase (XO), and uncoupled NOS. O_2_^•−^ is dismutated by superoxide dismutase (SOD) enzymes to hydrogen peroxide (H_2_O_2_), which can either be detoxified to water by catalase or be converted to the hydroxyl radical (^•^OH) in the presence of metal (iron-mediated Fenton reaction). NO^•^ and O_2_^•−^ spontaneously and rapidly react to form the strong oxidant peroxynitrite (ONOO^−^). ONOO^−^ can be detoxified by isomerization to nitrate (NO_3_^−^) or may form secondary radicals through homolytic fission (rupture of a covalent bond) or through reaction with carbon dioxide (CO_2_) of its conjugated acid peroxynitrous acid (ONOOH), yielding the carbonate radical (CO_3_^•−^), the nitrogen dioxide radical (NO_2_^•^) or the ^•^OH radical [9]

The term “reactive species” includes chemical substances, mainly reactive oxygen species (ROS) and reactive nitrogen species (RNS), formed upon incomplete reduction of oxygen [1] (Table 1 and Fig. 1). Precisely, when cells use oxygen, the redox process generates radical and not radical species as ROS and RNS, which appear to play a critical involvement in cellular signaling, cell growth, differentiation and, along with reactive halogen species, in cell immune response to infection by microorganisms [2, 3]. However, at high concentrations, they can cause cell damage and assist the development of diseases such as cancer, cardiovascular diseases, insulin resistance, diabetes mellitus and neurodegenerative disorders [4, 5]. Therefore, a constant level of reactive species can act as a message to control physiological processes, while excessive reactive species production can cause tissue malfunction or cell death. For these reasons, research into the regulation of reactive species level, mainly ROS, has attracted great interest, especially leading to the development of ROS-regulating therapies, like antioxidant therapies elicited by ROS scavengers and ROS-based cytotoxic therapies mediated by ROS-generating agents [6, 7]. Furthermore, over the past few decades, in the research on ROS regulation, nanotechnology has been specifically introduced with remarkable achievements thanks to the intrinsic physicochemical properties of nanomaterials, such as their tunable sizes (typically 10–100 nm), peculiar surface area/volume ratio and multifaceted interface/surface options [8].

**Table 1:** Reactive species

Reactive oxygen species (ROS)		Reactive nitrogen species (RNS)	
Name	Symbol	Name	Symbol
*Radicals*			
Hydroxyl	^•^OH	Nitric oxide	NO^•^
Superoxide	O_2_^•−^	Nitrogen dioxide	NO_2_^•^
Alkoxyl radical	RO^•^		
Peroxyl radical	ROO^•^		
*Non-radicals*			
Hydrogen peroxide	H_2_O_2_	Peroxynitrite	ONOO^−^
Singlet oxygen	^1^O_2_	Nitrosyl cation	NO^+^
Ozone	O_3_	Nitrosyl anion	NO^−^
Organic peroxide	ROOH	Dinitrogen trioxide	N_2_O_3_
Hypochlorous acid	HOCl	Dinitrogen tetraoxide	N_2_O_4_
Hypobromous acid	HOBr	Nitrous acid	HNO_2_
		Peroxynitrous acid	ONOOH
		Nitryl chloride	NO_2_Cl

Nanotechnology refers to research and development of structures with length scales between 1 and 100 nm strictly controlled at atomic, molecular and macromolecular levels [10]. Materials at this scale acquire new features and functions that vary significantly from those at the bulk scale. Indeed, the unique characteristics of these nanomaterials allow them to interact with complex biological functions in new ways, working at the same scale of biomolecules. This interaction has led multidisciplinary researchers to create multifunctional nanomaterials capable of targeting, diagnosing and treating diseases in a rapidly expanding discipline, termed nanomedicine [11]. In this regard, ROS-based therapeutic approaches, in which the depletion or generation of ROS can exert therapeutic effects, is one of the most interesting and promising field of nanomedicine referred as ROS-based nanomedicine [12]. Therefore, a huge range of nanomaterials with distinct ROS-regulating features have been developed for facilitating the chemical reactions involved in ROS for a wide range of medicinal applications such as the treatment of cancer, neurodegenerative diseases and bacterial infection. For these reasons, motivated by the outstanding advancements in this field, also confirmed by a rapid growth in the number of articles on this subject where ROS-based cytotoxic therapy seems the dominant research topic, a review of the main therapeutic strategies taking place to fight diseases thanks to nanotechnology-mediated ROS generation appears, in our opinion, useful and necessary.

**Fig. 2:**
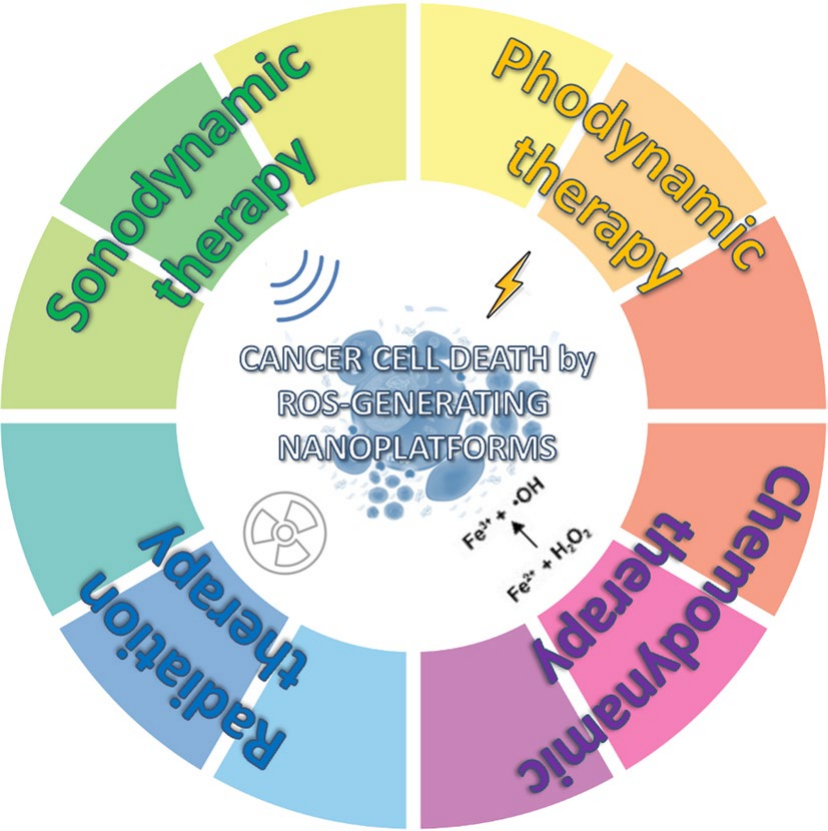
Schematic representation of anticancer approaches exploiting ROS-generating nanoplatforms

In this review we will discuss about those ROS-generating nanoplatforms that may be considered as front-runners for efficiently upregulating the intracellular redox status, in particular, focusing our attention on those tuned by interventions at the target site in cancer. Therefore, ROS-scavenging nanoplatforms but also ROS-mediated controlled drug release are out of the scope of this review since these nanosystems have been comprehensively reviewed elsewhere [12, 13]. This review aims at pointing out the foremost ROS-generating nanoplatforms designed to overcome the main limitations and improve the efficacy of anticancer ROS-based cytotoxic therapies. In particular, the ROS-generating nanoplatforms will be classified according to the strategy exploited for their selective activation like photodynamic therapy (PDT), sonodynamic therapy (SDT), radiotherapy and chemodynamic therapy, focusing also on their possible clinical trial translation (Fig. 2). Finally, the main methods to detect in vivo ROS production will be also discussed.

## ROS-based cytotoxic therapies

ROS are highly reactive molecules that can damage cellular components, therefore ROS-based cytotoxic therapies refer to treatments able to increase the intracellular level of ROS in order to elicit cellular stress and consequently to trigger cellular death. Indeed, harmful ROS can result in the formation of new chemical bonds, due to the presence of an unpaired electron in their outermost shell, which can lead to modification or damage of cell molecules like lipids, proteins, and DNA [14].

ROS can cause damage to lipids by oxidizing them and, as a consequence, leading to the formation of lipid peroxides. These peroxides can in turn cause damage to other biomolecules, such as proteins and DNA. ROS can also directly attack and modify proteins by oxidizing amino acid residues, which can alter their function or cause them to be degraded. ROS can also damage DNA by breaking the sugar-phosphate backbone or by forming mutations in the genetic code [15]. As a result, the production of excessive levels of ROS in the target sites has been used as a therapeutic strategy mainly against cancer and bacterial infection [16, 17]. This approach has encouraged the development of various ROS-generating nanoplatforms for upregulating the cellular redox status where, stimuli such as light, ultrasound (US) and nanozymes possessing peroxidase-like activities, are able of converting optical, mechanical and chemical energy, respectively, into intracellular ROS-based chemical energy possessing unique physicochemical properties. As follow, we will provide systematic explanations of the interdependence between the main features of ROS-generating nanoplatforms and their therapeutic actions in in vivo cancer models.

### ROS-generating nanoplatforms for cancer treatment

In cancer treatment, ROS-based cytotoxic therapies can be effective to selectively kill cancer cells, as malignant cells are often more sensitive to oxidative stress than healthy cells [18]. By inducing the accumulation of ROS in cancer cells, this approach can provoke cancer cell damage and trigger cancer cell death. Some examples include: (i) photodynamic therapy (PDT), a treatment that uses light and a photosensitizing agent or photosensitizer to generate ROS within cancer cells [19]: (ii) sonodynamic therapy (SDT), a developing anticancer approach, that utilizes US to trigger suitable compounds called sonosensitizers to initiate ROS production in cancer cells [20]: (iii) radiation therapy (RT), a mainstream cancer therapeutic modality that takes advantages of ionizing radiation to induce cell death by triggering death signaling in cancer cells through ROS generation and DNA damage [21], and (iv) chemodynamic therapy (CDT), an innovative cancer therapeutic approach that recruits Fenton or Fenton-like reactions to produce highly toxic ^•^OH in the tumour region [22].

In this section, we will discuss, for each cancer therapeutic option mentioned above, the most representative ROS-generating nanoplatforms able to overcome the main limitations, improving the efficacy of ROS-based cytotoxic therapies. In addition, a schematic table (Table 2) of each ROS-generating nanoplatform based on the external stimuli described is introduced to guide the reader throughout the review.

**Table 2:** ROS-generating nanoplatforms

Nanoplatform	Stimulus	Model	References
*ROS-generating nanoplatform and PDT*			
TiO_2_-coated UCNP core/shell nanocomposites (UCNPs@TiO_2_)	NIR and UVA irradiation	HeLa subcutaneous tumor-bearing Balb/c nude mice	[23]
UCNPs and black phosphorus sheets (UCNPs-BPS)	NIR laser	Tumor-bearing Balb/c mice obtained by subcutaneous injection of U14 cervical cancer cells	[24]
Cu_2_−xSe nanoparticles	NIR laser	Orthotopic glioma	[25]
Nanotraditional Chinese medicine system (TP + A)@TkPEGNPs)	Visible light	4T1-bearing mice	[26]
*ROS-generating nanoplatform and SDT*			
MnOx component with hollow mesoporous organosilica nanoparticles (HMONs) that are conjugated to protoporphyrin IX (PPIX), used as sonosensitizer, and arginine-glycine-aspartate (RDG)	US	Osteosarcoma tumor-bearing mice	[27]
3-bromopyruvate (BP) to PEGylated HMONs loaded with hematoporphyrin monomethyl ether (HMME@HMONs-3BP-PEG (HHBP)	US	4T1 tumor-bearing mice	[28]
alkyl radical generator 2,2-azobis[2-(2-imidazolin-2-yl)propane dihydrochloride (AIPH) onto a zirconium metal–organic framework (Zr-MOF)	US	Pancreatic cancer	[29]
*ROS-generating nanoplatform and radiation therapy*			
UCNP@silica nanostructure	X-ray radiation	Zebrafish	[30]
UCNP@silica nanostructure modified with polyethylene glycol and loaded with S-nitrosoothiol (PEG-USMSs-SNO)	X-ray radiation	4T1 mammary carcinoma-bearing mice	[30]
PEGylated (PEG) Au nanoparticles (RPAuNPs) functionalized with dihydrorhodamine 123 (DHR-123)	X-ray radiation	Xenograft mouse model of breast cancer	[31]
ultra-small Au NPs modified with responsive peptide (Tat-R-EK) consisting of three build blocks (Au@Tat-R-EK NPs)	X-ray radiation	LM3 liver tumor bearing nude mice	[32]
4-carboxybutyl triphenylphosphonium bromide (TiO_2_ (Gd)-TPP NPs)	X-ray radiation	MCF-7 xenograft tumor-bearing mice	[33]
*ROS-generating nanoplatform and chemodynamic therapy*			
PEGylated iron-engineered mesoporous silica nanoparticle (PEG/rFeOx-HMSN)	Fenton-based reactions	4T1 mammary tumor-bearing mice	[34]
ferrous-cysteine–phosphotungstate nanoparticles (FcPWNPs)	Fenton-based reactions	4T1 tumour-xenografted BALB/c	[35]
Glucose oxidase (GOD) and Fe_3_O_4_ NPs were incorporated into biodegradable silica NPs (GOD-Fe_3_O_4_@DMSNs)	Fenton-based reactions	4T1 breast cancer xenografts and U87 tumour xenografts	[36]
Core–shell Mn3[Co(CN)6]2@MIL-100(Fe) metal–organic frameworks (CS-MOFs) nanocubes	Fenton-based reactions	HeLa-tumor-bearing mice	[37]
Linoleic acid hydroperoxide (LAHP) nanoplatform (IO-LAHP)	Fenton-based reactions	U87MG tumour	[38]

### Photodynamic therapy

The basic principle of PDT consists in an exogenous light-mediated release of energy for triggering a harmless photosensitizer inside target cells to transfer its excited-state energy to oxygen (O_2_) [39]. This can cause malignant cells to die through apoptosis and/or necrosis, and it can also stimulate the host immune system [40]. As a result, photosensitizer and tissue oxygen are PDT's key components, and usually the photosensitizers are subjected to an excitation by short-wavelength ultraviolet (UV)-visible light, which features poor tissue penetration. Therefore, in recent year, some groundbreaking photosensitizers based on nanomaterials have been developed to overcome such limitation. Typically, upconversion nanoparticles (UCNPs) are able to convert near-infrared (NIR) to visible light, which in turn activates an organic/inorganic photosensitizer by the transfer of electronic excitation energy either through absorption of upconversion luminescence photon by the photosensitizer or via fluorescence resonance energy transfer (FRET) [41–43].

**Fig. 3:**
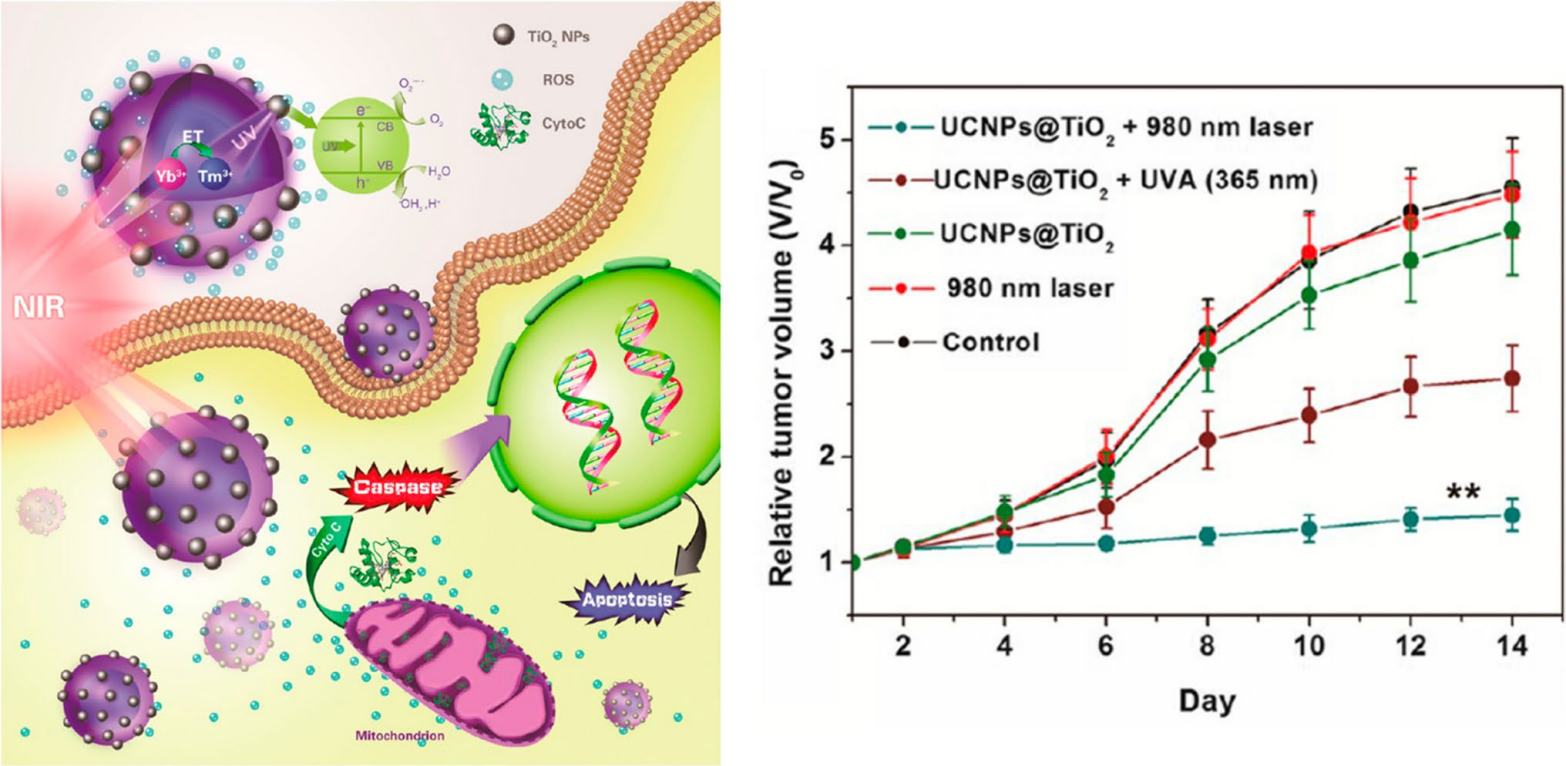
Graphical representation for the potential molecular mechanism of PDT mediated by UCNPs@TiO_2_ and NIR irradiation, and in vivo tumor volume changes in tumor-bearing mice of different treatment groups (n = 6, **p < 0.01 compared with the control group) [23]

Most of the studies on UCNPs have been performed on in vitro cell cultures, being the main aim to demonstrate the uptake of PS-loaded UCNPs and the cell death following NIR irradiation. Surprisingly, considering all the in vivo studies, to the best of our knowledge, only one work has tried to answer the central question of whether the use of UCNPs extends the tissue depth at which PDT can be effective [44, 45]. Indeed, Hou and colleagues are the only one that compared the in vivo anticancer effects of their nanosystem irradiated by NIR and UV-A laser, respectively [23]. Briefly, the authors have designed an innovative NIR activated photosensitizer for PDT made by TiO_2_-coated UCNP core/shell nanocomposites (UCNPs@TiO_2_). First, the authors investigated the cellular uptake and the intracellular location of UCNPs@TiO_2_ in HeLa cells providing direct evidence that the uptake of UCNPs@TiO_2_ occurs by endocytosis and that UCNPs@TiO_2_ mainly accumulates in lysosomes or around mitochondria. Then, cytotoxicity of UCNPs@TiO_2_ on HeLa cells was investigated under 980 nm irradiation showing a remarkable viability decrease compared to those exposed to visible light irradiation or UCNPs@TiO_2_ alone. In particular, the phototoxicity of UCNPs@TiO_2_ NCs resulted in cytotoxicity of HeLa cells thanks to the ROS formation proved by intracellular conversion of nonfluorescent 2,7-dichlorofluorescin diacetate (DCFH-DA) to fluorescent dichlorofluorescein (DCF). Moreover, the pathway of cell death induced by UCNPs@TiO_2_ under NIR irradiation was also investigated suggesting apoptosis as the main modality due to disruption of mitochondrial activity and activation of caspases (Fig. 3).

To study the therapeutic in vivo potential, HeLa subcutaneous tumor-bearing Balb/c nude mice were intratumorally injected with UCNPs@TiO_2_ and the tumor sites were irradiated by NIR and UV-A laser, respectively. The group treated with NIR-irradiated UCNPs@TiO_2_ showed a significant tumor growth inhibition compared to the groups that received intratumorally injected saline, UCNPs@TiO_2_ without NIR laser exposure and UV-A-irradiated UCNPs@TiO_2_. The latter comparison confirmed that the suggested approach provides an encouraging alternative to overcome the poor tissue penetration of UV-A light, that is the main limitation of current PDT (Fig. 3). Furthermore, thanks to the Gd and Yb ions in the UCNPs@TiO_2_, these core shell nanocomposites could be used as potential MRI and CT contrast agents to monitor their in vivo pharmacokinetics/ pharmacodynamics. Furthermore, these nanocomposites possessing efficient anticancer photodynamic activity and useful imaging capability place themselves as an encouraging multifunctional anticancer nanoplatform to bioimaging and therapeutics simultaneously.

Even if significant achievements have been reached in PDT thanks to UCNPs, their therapeutic efficacy is still not suitable enough to completely fulfil the strict requisites of clinical translation. Although this strategy seems to overcome one of the main limitations of PDT, the overheating effect of laser irradiation at 980 nm, widely used to excite UCNP, could jeopardize its further development. Therefore, novel UCNPS have been studied to successfully shift the excitation of UCNPs from 980 to 808 nm, being the heating effect minimized and the tissue transparency maximized [46, 47]. In this regard, Lv et al. designed a novel multifunctional composite of UCNPs and black phosphorus sheets (UCNPs-BPS) to achieve the 808 nm NIR-mediated PDT [24]. The in vivo antitumor performance of the nanoplatform was evaluated on tumor-bearing Balb/c mice obtained by subcutaneous injection of U14 cervical cancer cells. The group treated with UCNPs-BPS and 808 nm NIR-light irradiation showed the strongest tumor growth inhibition, while 808 nm NIR-light irradiation alone had a negligible effect as the treatment with UCNPs-BPS alone. However, the authors showed the high anticancer efficiency of UCNPs-BPS under 808 nm NIR-light irradiation but did not prove a superior effect compared to UCNPs-BPS under UV-A irradiation to support its ability to go beyond the poor tissue penetration of conventional PDT. According to this work, the authors proved that the UCNPs-BPS composite elicits ROS generation when irradiated with 808 nm NIR light in a greater extent than under 650 and 980 nm irradiation, determining improved anticancer results; at the same time, a real-time monitoring could be performed by using the emitted green light [24].

Since a characteristic of NIR laser irradiation could be to increase the Fenton-like reaction, Wang and colleagues introduced a high-performance biomimetic nanocatalyst, where glucose oxidase (GOD) was conjugated to Cu_2_–xSe nanoparticles, and encapsulated within tumor cell membranes [25, 48]. The glucose oxidation provoked by the nanocatalyst in the tumour is able to boost the Fenton-like reaction determining an increase in H_2_O_2_ level. The in vivo anticancer experiment has been developed by using groups of mice bearing the 4T1 tumors. When the concentration of H_2_O_2_ reached the highest level, a laser at 1064 nm was used to irradiate the tumor thanks to its deep penetration. The combination of laser irradiation and H_2_O_2_ increased the rate of Fenton reaction and the massive production of ^•^OH, resulting in a successful tumor therapy. The authors were therefore able to demonstrate that the Cu_2_–xSe NPs showed a strong responsiveness to laser irradiation for the PDT of deep orthotopic glioma. They also showed that the use of a laser in the NIR II region at 1064 nm provoked a much stronger anticancer effect, compared to the use of a laser in the NIR I window at 808 nm. Therefore, their nanosystem could be used for the treatment of other tumors deeply seated [25].

In another work, Zhang and colleagues [26] developed an innovative nanosystem based on a traditional Chinese medicine, able to remodel autophagy homeostasis (TP+A)@TkPEGNPs). In this ROS-generating nanosystem, a photosensitizer aggregation inducing emission (AIE) was encapsulated with an autophagy modulator triptolide (TP, an active ingredient of Tripterygium wilfordii Hook F). The antitumour effect under light exposure was investigated in an in vitro system, evaluating the effective ROS production, and in an in vivo model of triple negative breast cancer using 4T1-bearing mice. Authors showed a statistically significant induction of the autophagy pathway with efficient anticancer activity, therefore this naosystem could open a new way to manage the autophagy homeostasis remodeling [26].

### Sonodynamic therapy

Thanks to its non-invasiveness, non-ionizing and tissue penetrating properties, US has been widely studied as an external source of activation in various therapeutic applications, such as high intensity ultrasound (HIFU)-mediated hyperthermia, US-triggered drug release and sonodynamic therapy (SDT) [49–52]. In particular, SDT exploits US for activating suitable compounds (sonosensitizers) to trigger ROS generation in cancer cells leading to cancer elimination [20]. Compared to PDT, SDT could have a wider application in cancer treatment, as the tissue penetrability of US allows SDT to reach even deep cancer tissues, thus overcoming one of the main limitations of PDT.

Despite growing interest, the mechanisms underlying the therapeutic activity of US have not yet been clearly elucidated, slowing the clinical application of SDT. At least three mechanisms are involved and some or all of them may occur simultaneously during US exposure, depending on the physicochemical characteristics of the sonosensitiser and its localization at cellular level [20].

To summarize, it has been hypothesized that these mechanisms, i.e., sonochemical effects, sonomechanical effects and sonoluminescence (SL), rely on acoustic cavitation resulting from the interaction between liquid medium and US where, at least for the last two mechanisms, ROS production must occur to kill tumour cells. Since SDT is one of the biomedical fields that has significantly improved in recent years thanks to increased knowledge of nanotechnology, we believe that investigating the role of SL as an internal light source for the photoactivation of advanced nanoplatforms represents a real step forward in SDT development and beyond [31, 53].

SL refers to the transformation of the mechanical energy of into light pulses of about 35–350 picoseconds and that are composed of huge amount of photons, between 3 × 10^4^ and 3 × 10^5^ [54]. SL is supposed to induce a progressive activation a photochemical reaction similar to the one of PDT, therefore provoking sonosensitizers excitation from the ground state to a short-lived excited single state. Therefore, the resultant triplet state can react with the substrate generating free radicals (e.g., ^•^OOH, ^•^OH and O_2_^•−^) via a type I photodynamic reaction, by transferring electrons. Otherwise, the free radicals can directly react, via energy transfer, with O_2_ to generate ^1^O_2_ via a type II photodynamic reaction [39]. Since an early work by Umemura and colleagues [55], numerous researchers have studied SL and ^1^O_2_ generation in vitro and in vivo. However, a general consensus has not yet been formulated. Recently, Giuntini and colleagues have studied the role of SL in SDT analyzing the US responsiveness of a variety of porphyrin complexes, namely metal-free porphyrin and Fe(III), Pd(II) and Zn(II) porphyrins, in terms of ROS production under US stimulation [56]. The SL emissions was detected in the UV/visible range of the electromagnetic spectrum, therefore allowing to support the hypothesis that SL can be responsible for the SDT activation of porphyrins.

However, even if some in vitro studies have demonstrated the potential role of SL in SDT, there is no information, so far, regarding SL activity in vivo, maybe for technical reasons, to support the SL hypothesis in SDT. To overcome the difficulties to measure SL in vivo, some attempts were carried out in models mimicking human tissue properties. This is the case of Sazgarnia and colleagues, who developed a mimicking tissue material based on agar where a fiber optic with special connectors was used for light transfer from the tissue mimicking material to the spectrometer to investigate the protoporphyrin conjugated to gold NPs under US exposure [57]. The authors were able to measure SL and ^•^OH, giving some evidence of the ROS-based sonodynamic mechanism via SL. Unfortunately, no more investigations from these authors but also from other researchers have been performed to confirm the presence of SL in vivo, therefore the role of SL in SDT in vivo still remains elusive.

Being SDT a ROS-based cytotoxic therapy, some researchers have tried to increase its efficacy, independently from the main mechanisms of action involved, developing nanosystems to increase ROS production by (i) tackling hypoxia or (ii) taking advantage of the tumor hypoxia to enhance cancer cell damage [13]. In particular, hypoxia occurs in tumor sites characterized by low oxygen supply and induces biological responses that hamper the therapeutic outcomes of a variety of anticancer approaches [58]. Moreover, SDT can lower oxygen content as it uses molecular oxygen to induce the US and sonosensitizer-mediated ROS generation.

The co-delivery of O_2_ and sonosensitizers has been exploited in nanoformulations that combine sonosensitizers and oxygen-carrying perfluorocarbons in tumor hypoxic tumor microenvironment (TME) [13, 59]. Another successful strategy to increase the oxygen amount at tumor levels, involves the development of nanoplatforms by combining a MnOx component with hollow mesoporous organosilica nanoparticles (HMONs) that are conjugated to protoporphyrin IX (PPIX), used as sonosensitizer, and arginine-glycine-aspartate (RDG), used as targeting peptide. The nanoplatform was designed to modulate TME hypoxia and to consequently augment the sonodynamic efficacy of the sonosensitizer when US exposure was perfomed. MnOx NPs were produced in the mesopore channels of HMONs in a redox reaction to act then as a nanoenzyme catalyzing the overexpressed H_2_O_2_ in the TME for oxygen production. The nanoplatform was deeply investigated both in vitro, in the human U87 glioblastoma cell line, and in vivo, in U87 tumor-bearing mice, and significant cancer cell killing and tumor growth suppression under US exposure was observed [27]. HMONs have also been loaded with PPIX, used as a sonosensitizer, and integrated with ferrate (VI) to produce O_2_ via the ferrate (VI)-mediated decomposition of H_2_O and H_2_O_2_. Enhanced sonodynamic treatment efficacy was demonstrated in a model of osteosarcoma, proving that contemporaneous oxygen generation, in situ GSH depletion, and ROS overproduction play a synergistic role in stimulating the sonodynamic treatment toward hypoxic solid tumors. These findings support promising SDT applications also for the treatment of hypoxic solid tumors [60].

Hypoxia-responsive nanovesicles have recently been developed as delivery vehicles for releasing the sonosensitizer and, at the same time, generating oxygen at the tumor site. In a hypoxic TME, the dissociated nanovesicles were able to release 5-aminolevulinic acid (5-ALA), then converted into the sonosensitizer PPIX, and the embedded manganese ferrite NPs that catalyzed H_2_O_2_ into oxygen resulting in enhanced SDT efficiency, that was demonstrated on B16 melanoma-bearing mice [61].

**Fig. 4:**
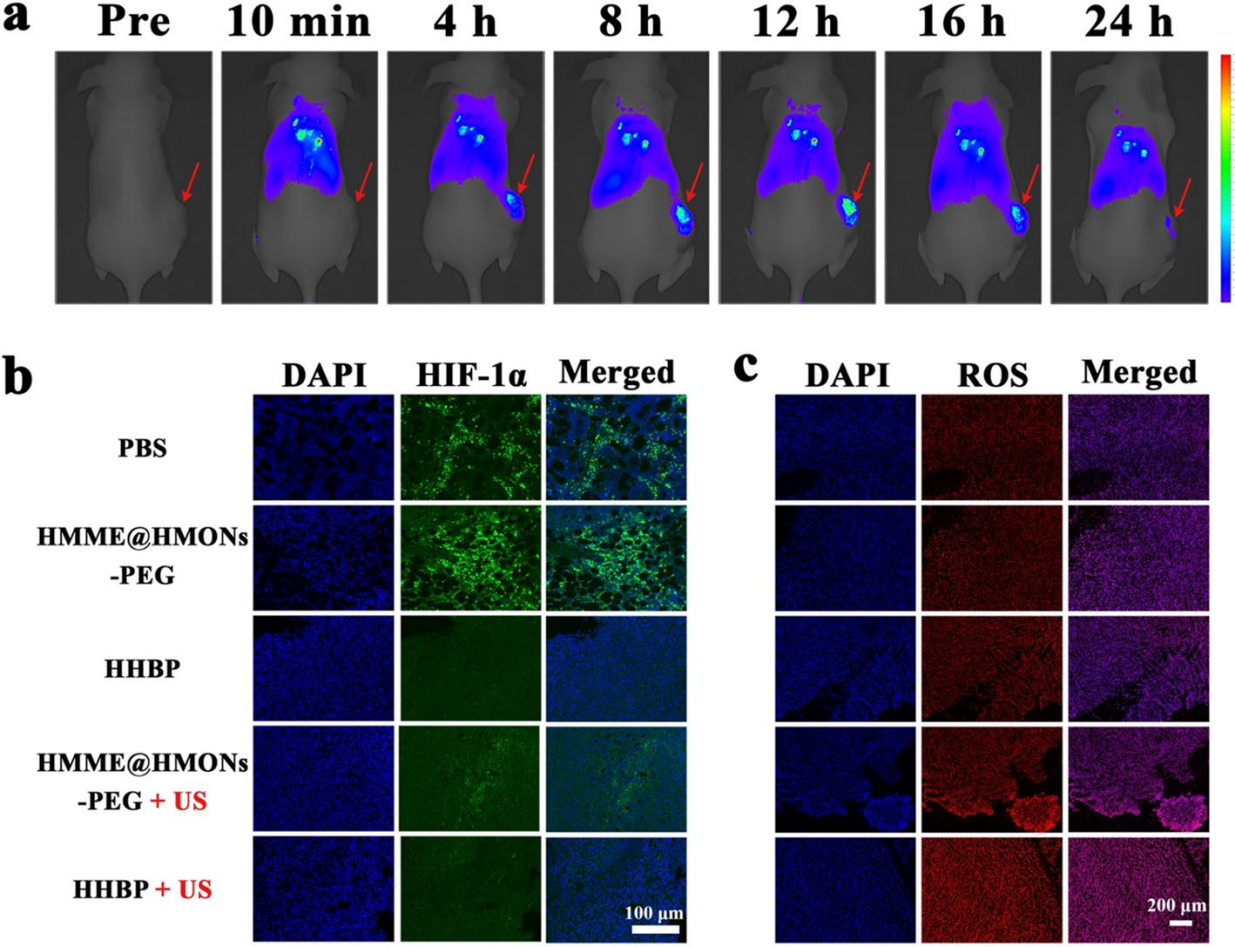
In vivo biodistribution of the HHBP and the alleviation of hypoxia. **a** In vivo fluorescence image of 4T1 tumor-bearing mice at different time points after intravenous injection of ICG-loaded HHBP. The red arrows indicate the tumor sites. **b** HIF-1α staining tumor tissues harvested from 4T1 tumor-bearing mice after different treatments. **c** Fluorescence images of tumor slices were collected at 24 h after different treatments and staining with the ROS probe, dihydroethidium (DHE, red) [28]

Interestingly, rather than using coping strategies to amplify the O_2_ amount at tumor site, a biodegradable O_2_ economizer has been prepared for enhancing SDT efficacy by suppressing O_2_-consumption and triggering a pro-death autophagy strategy. The O_2_ economizer consists of the conjugation of the respiration inhibitor 3-bromopyruvate (BP) to PEGylated HMONs loaded with hematoporphyrin monomethyl ether (HMME), used as sonosensitizer, namely HMME@HMONs-3BP-PEG (HHBP). The increased sonodynamic treatment efficacy was verified under US exposure in both in vitro and in vivo models (Fig. 4) [28]. Thanks to this work, the authors proposed an O_2_-economizer strategy to reverse the tumor hypoxia and increasing the SDT therapeutical efficacy, as an alternative for the ongoing mainstream hypoxia-regulation precept of elevating intratumoural O_2_ level. Furthermore, this work lays a new alternative method for manipulating the autophagic processes in order to increase the efficacy of ROS-mediated cancer therapy [28].

Another strategy consists in taking advantage of the tumor hypoxia induced by the sonodynamic activity to enhance cancer cell damage. Hollow mesoporous TiO_2_ NPs with modified S-nitrosothiol (SNT) and loaded with tirapazamine (TPZ) have been developed to this end. When the nanosystem was exposed to US at tumor site, the induced ROS generation was able to sensitize the SNT groups that then released the NO that was responsible for an increased anticancer sonodynamic effect, mainly by increasing mitochondria damage. Moreover, the hypoxic TME that resulted from the oxygen consumption caused by the sonodynamic activity of TiO_2_ was able to activate the cytotoxicity of TPZ, leading to increased cancer cell DNA damage [62]. Indeed, this versatile all-in-one nanotheranostic system was considered a valid candidate to provide US-mediated tritherapies and US imaging of cancer simultaneously, which offered compelling marks for advancing nanotherapy in biomedical field application. Indeed, the functionalization of the NPs surface showed an increased performance in vivo, even if the effects of NO in a dose-dependent pharmacological was need to be deeply investigated as clearly stated by the authors.

Finally, a dual-sonosensitizer nanoplatform has been developed by loading an alkyl radical generator 2,2-azobis[2-(2-imidazolin-2-yl)propane dihydrochloride (AIPH) onto a zirconium metal–organic framework (Zr-MOF) to enhance SDT under hypoxic condition. Under US exposure, the NPs were able to efficiently produce ^1^O_2_ in normoxic conditions, as well as alkyl radicals in both normoxic and hypoxic conditions, leading to cancer cell death. Therefore, the NPs guaranteed significant sonodynamic anticancer effects under both normoxic and hypoxic conditions under US exposure in in vitro and in vivo pancreatic cancer models [29]. Furthermore, in this work, the biocompatibility, and imaging ability of Zr-MOF@AIPH, along with the good photoacoustic, fluorescence and US imaging characteristics thanks to their porphyrin-based structure and the nitrogen generated, have been showed. This gives the authors the possibility to move toward an improvement of SDT efficacy in a condition of hypoxic environment by combining together complementary sonosensitizers [29, 63].

### Radiation therapy

Radiotherapy (RT) uses ionising radiation to trigger aberrant physicochemical changes in tumour cells by the generation of ROS, stress response in subcellular organelles, like mitochondria and endoplasmic reticulum, and DNA damage [21].

During radiotherapy ROS are generated by H_2_O ionization in tumour tissue, either through the irradiation from external radiation beams (e.g., high-energy X ray) or internal radioisotopes, being these highly reactive agents toxic toward cancer cells and adjacent healthy tissues [64]. Moreover, radiation can stimulate ROS generation in mitochondria with redox status imbalances leading to oxidative stress via reaction with proteins, lipids, and DNA to cause protein misfolding, lipid peroxidation and DNA strand breaks [21, 65, 66]. For these reasons in oncology, RT is a potent strategy to enhance intracellular ROS levels and to consequently induce cancer cell death, mainly in a p53-dependent manner [21].

Despite the many advantages of RT in cancer treatment, like the possibility of treating different tumour types, alleviating cancer pain and being combined with other anticancer treatments, RT is a challenging treatment as it might damage healthy cells and cause the development of cancer cell radioresistance, tumour recurrence and the development of other types of cancers [21, 67–69]. To improve the therapeutic effect of ionizing radiation researchers mainly aim at confining the radiation dose to the tumour volume and at improving tumor tissue radiosensitization [29].

Several approaches to localize the radiation dose to the tumor volume have been developed including (i) image-guided radiation therapy (IGRT), (ii) intensity-modulated radiation therapy (IMRT), (iii) volumetric modulated arc therapy (VMAT) and (iv) stereotactic body radiation therapy (SBRT) [70–73]. On the other hand, to improve cancer cell radiosensitization nanotechnology has attracted strong interest thanks to nanomaterials possessing intrinsic radiosensitive activities as increased ROS generation. In this section, we will focus on recent advances in the development of ROS-based nanoradiosensitizers for improving therapeutic outcomes at low-dose ionizing radiation.

**Fig. 5:**
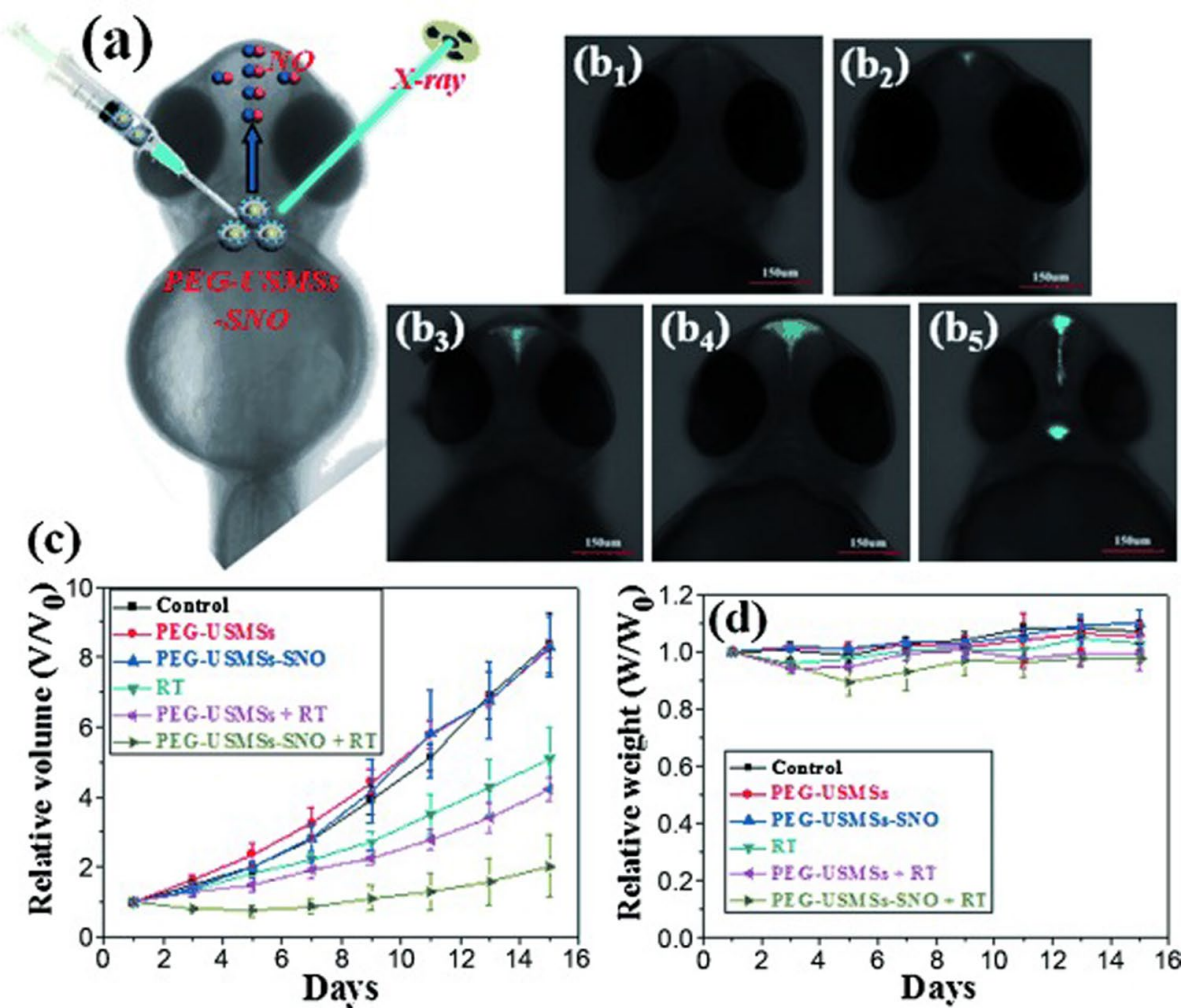
**a** X‐ray‐triggered NO release from PEG‐USMSs‐SNO in zebrafish larvae. **b** Confocal laser scanning microscope (CLSM) images of NO release in living zebrafish larvae with brain ventricle microinjection of PEG‐USMSs‐SNO upon exposure to different doses of X‐ray radiation: b_1_ 0 Gy, b_2_ 1 Gy, b_3_ 3 Gy, b_4_ 5 Gy, b_5_ 10 Gy. **c** Relative tumor growth curve and **d** weight change curve of 4T1 tumor‐bearing mice over half a month after different treatments [30]

Nanoradiosensitizers, when combined with radiation, are able to exert a greater tumour-inactivating effect than radiation alone and can be classified according to the nanomaterials involved such as nanomaterials with high Z-elements (Z represents the material’s atomic number) or releasing nitric oxide (NO) under X-ray radiation [74]. Indeed, recent works have shown that NO generation could sensitize radiation therapy by inhibiting the DNA repair and reacting with ROS to yield more toxic ONOO^−^ [12]. For example, Fan and colleagues developed a UCNP@silica nanostructure modified with polyethylene glycol and loaded with S-nitrosoothiol (PEG-USMSs-SNO), where the S–N bond can be cleaved by X-ray irradiation thanks to the ROS generation and release electrophilic NO to enhance the radiation therapy [30]. In in vivo, first zebrafish was selected for monitoring the NO release responsive to X-ray by confocal laser scanning microscope imaging confirming that PEG-USMSs-SNO can be suitable for the X-ray-controlled NO release in vivo (Fig. 5a, b). The authors then investigated NO-radiosensitizing effects on treating 4T1 mammary carcinoma-bearing mice. PEG-USMSs-SNO cause a significant inhibition of tumor growth over time, that should be attributed to a significant enhancement of radiation effects by the NO-radiation, compared to RT and PEG-USMSs + RT (Fig. 5c).

Finally, PEG-USMSs-SNO are biologically safe and provoke few side effects as no noticeable fluctuations in mice weight were observed (Fig. 5d).

Regarding nanomaterials with high Z elements, gold (Au) nanostructures (Z = 79) with high chemical inertness are the most representative radionanosensitisers that have been extensively investigated for improving radiation deposition at the tumour site [75–77]. The mechanism of radiosensitisation of Au nanoparticles is mainly achieved by following three key biological pathways: (1) production of ROS, oxidative stress and mitochondrial dysfunction; (2) effect on the cell cycle and (3) inhibition of DNA repair [78]. Other possible biological mechanisms have also been proposed, such as autophagy and ER stress. Focusing on the first biological mechanism, Klein and colleagues developed a nitrosyltetrafluoroborate (NOBF_4_) stabilized AuFe_3_O_4_ nanoheterodimers that under X-radiation led to the simultaneous production of NO and the O_2_^•−^ radicals that effectively form ONOO^−^ [79]. However, the destructive effectiveness of ONOO^−^ and other ROS was only evaluated on human MCF-7 breast cancer cells.

**Fig. 6:**
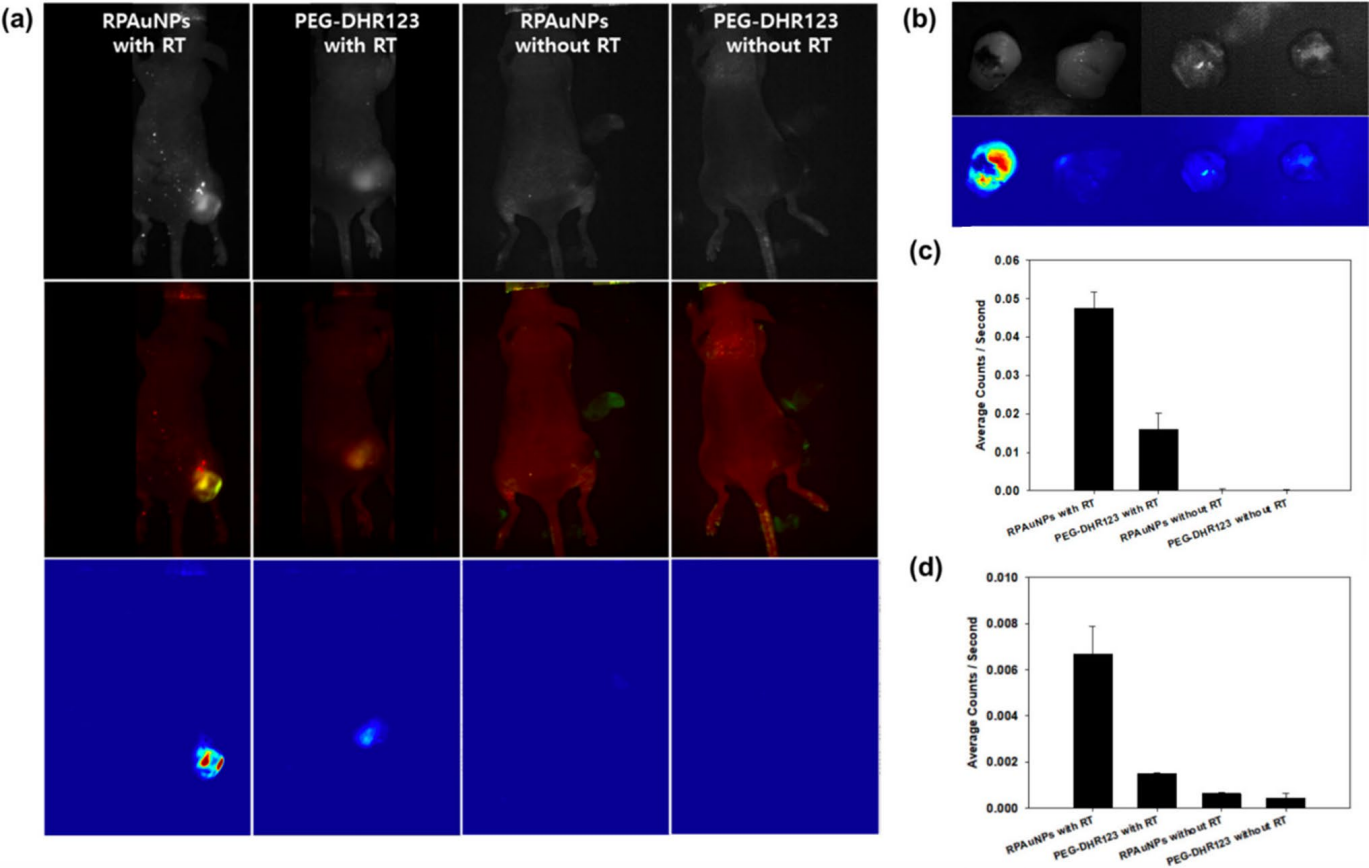
ROS generation and detection in vivo using RPAuNPs. **a** Top row Raw in vivo fluorescence images acquired with the tunable emission filter set to 530 nm, for mice injected with either RPAuNPs or PEG-DHR123, and either exposed to 6 Gy RT or no RT. **a** Middle row two-component images showing DHR123 fluorescence (green) and skin autofluorescence (red), obtained by spectral unmixing. **a** Bottom row DHR123 fluorescence, obtained by spectral unmixing. **b** Same tumors, imaged ex vivo, showing reflectance image (top) and unmixed fluorescence image for the DHR123 component (bottom). **c** Fluorescence counts obtained from unmixed DHR123 in vivo images. **d** Same as previous, for ex vivo images [31]

To investigate the role of Au nanostructures in generating ROS to sensitise cancer cells to radiotherapy in vivo, Choi et al. developed PEGylated (PEG) Au nanoparticles (RPAuNPs) functionalized with the ROS sensor dihydrorhodamine 123 (DHR-123) to prove ROS generation in a xenograft mouse model of breast cancer [31]. Figure 6 shows tumor-bearing mice where both RPAuNPs and PEG-DHR123 were injected intratumorally. A significant fluorescent signal was observed in irradiated tumors 1 h later multispectral fluorescence imaging (RPAuNPs with RT and PEG-DHR123 with RT) but not in non-irradiated tumors, i.e. control, (RPAuNPS without RT and PEG-DHR123 without RT) (Fig. 6a). The imaging of excised tumors ex vivo confirmed the results (Fig. 6b). Moreover, fluorescence activation was significantly higher in tumors injected with RPAuNPs with RT compared to control tumors injected with PEG-DHR123 with RT. The average photon counts determined by quantitative analysis in the irradiated RPAuNP tumors were higher compared to controls (Fig. 6c) as also confirmed in excised tumors (ex vivo), where the difference was even higher between irradiated RPAuNP tumors and control tumors (Fig. 6d). Therefore, the tumor targeted X-ray irradiation efficiently generates ROS.

**Fig. 7:**
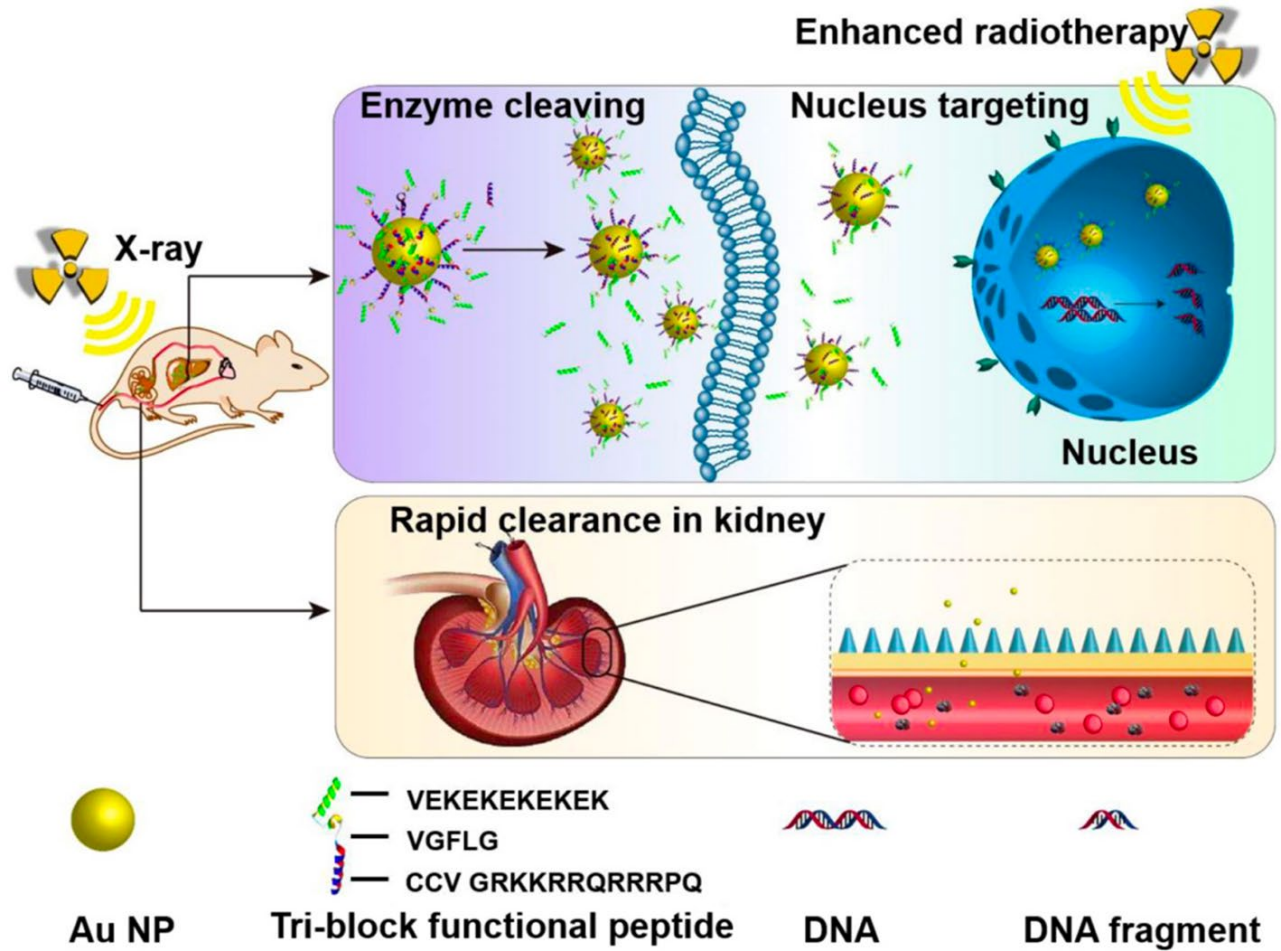
Schematic illustration of the accumulation in tumour tissues and cell nuclei for enhanced radiotherapy in vivo, and the rapid clearance via kidney tri-block functional peptides coated ultrasmall Au NPs. Tri-block functional peptides: VEKEKEKEKEK, a zwitterionic peptide sequence consisting of alternative glutamic Acid (E) and lysine (K) with great antifouling property in vivo; VGFLG, a peptide sequence which is known to be cleaved by overexpressed cathepsin B in the microenvironment of many different tumors; CCVGRKKRRQRRRPQ, a powerful cell penetrating and nuclear targeting peptide sequence, known as Tat peptide, derived from human immunodeficiency virus-1 (HIV-1) transactivator of transcription protein [32]

Another example in which, following X-ray irradiation of ultra-small Au NPs modified with responsive peptide (Tat-R-EK) consisting of three build blocks (Au@Tat-R-EK NPs), overgenerated ROS lead to protein oxidation, lipid peroxidation and DNA damage, which are supposed to be closely linked to tumour volume suppression and increased survival in orthotopic transplanted LM3 liver tumor bearing nude mice, was provided by Ding and colleagues (Fig. 7) [32]. The low dimension of Au NPs assures a harmony between rapid renal clearance and an effective tumour targeting and also a good biocompatibility and tumor radiotherapy outcome against orthotopic LM3 liver tumors [32].

**Fig. 8:**
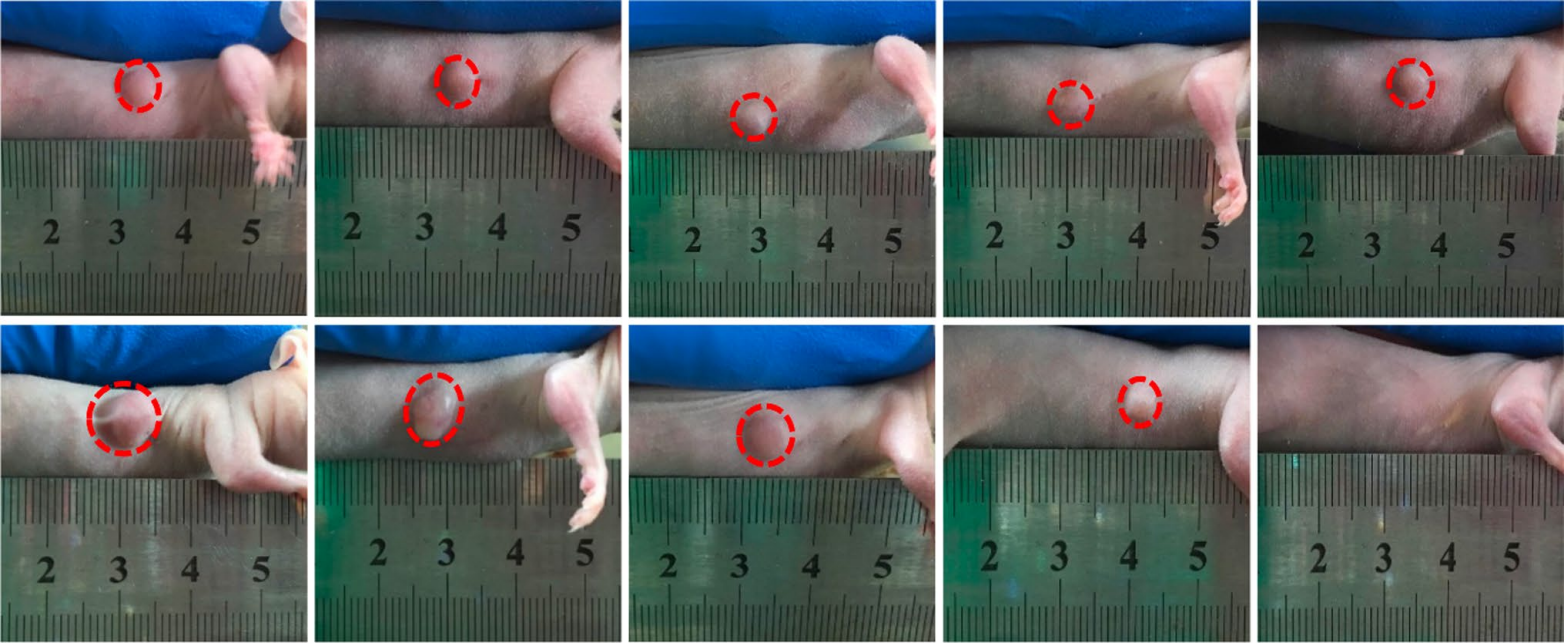
Photographs of the mice before (Day 0) and after (Day 14). The dosage of TiO_2_ (Gd)-TPP NPs was 3 mg kg^−1^, followed by X-ray irradiation (6 Gy) [33]

Moreover, it has been documented that many other nanomaterials with high Z elements also adopt their strong photoelectric absorption abilities for ROS-based RT treatment. An example comes from Chen and colleagues who developed a gadolinium-doped titania nanosensitizer funtionalized with 4-carboxybutyl triphenylphosphonium bromide (TiO_2_ (Gd)-TPP NPs) that, after irradiation with X-ray, targets mitochondria to accumulate ROS inside them, killing cancer cells [33]. The authors investigated whether the therapeutic effects of TiO_2_ (Gd)-TPP NPs, to elicit ROS increase in mitochondria, would improve RT-mediated cancer cell killing in in vivo animal model. For this reason, TiO_2_ (Gd)-TPP NPs were intratumorally injected into MCF-7 xenograft tumor-bearing mice and after X-ray radiation, the mice presented a significant tumor growth inhibition with complete tumor eradication at 14 days post-treatment (Fig. 8). The nanosensitizer combined toghether with a single X-ray radiation exposure resulted in a complete tumor ablation in a mouse model, without side effects during the treatment. The results showed that the mitochondria-targeted nanosensitizer could dramatically reduce treatment doses, while amplifying antitumor efficacy, and providing potentially a highly efficacious and universal approach to meliorate the tumor radiosensitivity for future clinical applications against cancer. Furthermore, it has been reported that iron-based nanomaterials facilitate ROS production under X-ray irradiation due to the iron-catalysed Haber–Weiss cycle and Fenton reaction [80].

### Chemodynamic therapy

The previously discussed cancer therapeutic modalities (PDT, SDT, RT) to trigger ROS formation are all based on external physical stimuli (UV–vis light, US and X-rays) that however entail certain disadvantages: limited tissue penetration (e.g., PDT), poorly understood mechanism of action (e.g., SDT), and unavoidable damage to normal tissue, (e.g., RT), which significantly compromise therapeutic results. Recently, scientific works have been moving towards new and very interesting anti-cancer therapeutic concepts to overcome such limitations, such as the exploitation of tumor microenvironment (TME) as an endogenous stimulus. Indeed, TME shows a mild acidity, a high concentration of GSH and a high level of H_2_O_2_, providing likely tools for an accurate selection between tumour and healthy tissues. For this reason, several studies have attempted to develop nanomaterials capable of enabling specific chemical reactions only within tumour tissue rather than in healthy tissue. In fact, these non-harmful nanomaterials should only turn into highly cytotoxic ROS within tumour tissues and, this ROS-generating tumor-specific therapeutic modality was termed as chemodynamic therapy (CDT) [22, 51, 81].

In this innovative and promising antitumour approach, the formation of highly cytotoxic ROS towards tumours by nanomaterials occurs because nanomaterials are primarily designed to respond to the acidic environment and high concentrations of H_2_O_2_ intratumourally to promote Fenton or Fenton-like reactions in the TME. Fenton described for the first time the so-called “Fenton reaction” consisting in the enhanced oxidative potential of H_2_O_2_, when iron (Fe) is used as a catalyst under acidic conditions [82]. In simple terms, the chain reaction between ferrous ion (Fe^2+^) and H_2_O_2_ catalyzes the formation of ^•^OH under acidic conditions. Besides Fe^2+^, other transition metal ions, such as cupric ion (Cu^2+^) and manganese ion and (Mn^2+^), can also catalyze similar chemical reactions, known as Fenton-like reaction [83].

In this section, we will focus on CDT where advanced nanomaterials with versatile metal ions e.g., Fe^2+^ Cu^2+^ and Mn^2+^ catalyze intracellular H_2_O_2_ for ROS generation in in vivo model. Therefore, chemodynamic approaches, based on the combination of CDT with light, US, electricity and chemotherapy to overcome the drawbacks of traditional Fenton oxidation and to improve the therapeutic effects are out of the scope of this review because they are extensively discussed in the existing scientific literature [22].

**Fig. 9:**
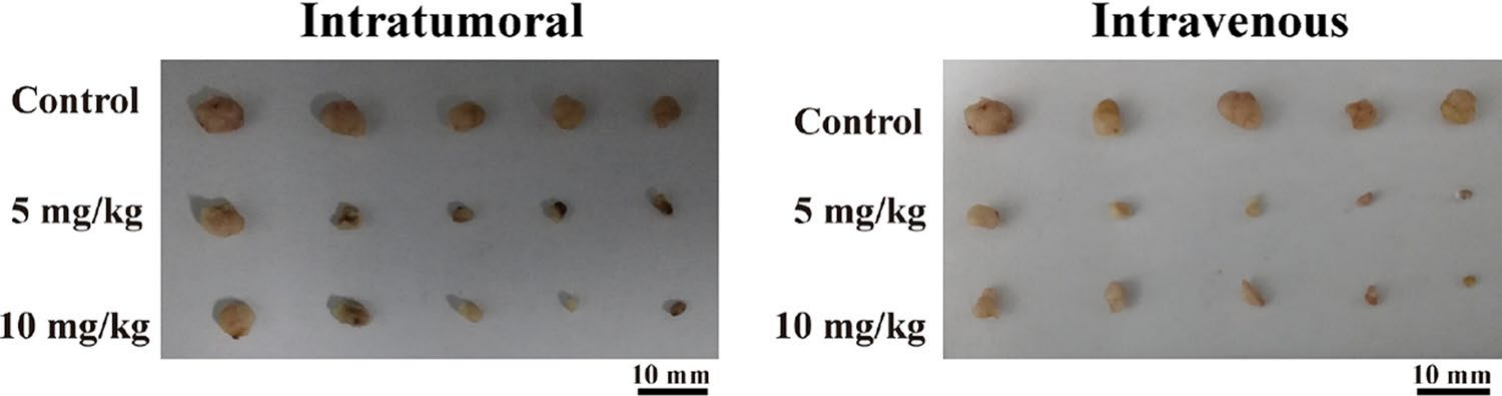
Digital photographs of dissected tumors after the therapeutic process from intratumoral and intravenous groups [34]

CDT can be achieved by exploiting inorganic, organic and inorganic–organic nanoplatforms. For example, Wang and co-authors developed a PEGylated iron-engineered mesoporous silica nanoparticle (PEG/rFeOx-HMSN) to realise a nanocatalyst, where the overproduction of H_2_O_2_ and the slightly acidic nature of the TME, could trigger Fenton-like reactions in situ leading to ^•^OH overproduction and consequently significant oxidative damage on 4T1 mammary tumor-bearing mice [34]. The PEG/rFeOx-HMSN nanocatalyst was injected intratumorally (5 mg/kg) and intravenously (10 mg/kg) onto 4T1 tumor-bearing mice. Importantly, the high therapeutic efficacy of the PEG/rFeOx-HMSN nanocatalyst was proved by the suppression of 4T1 breast tumour growth after both the ways of administration (Fig. 9). Furthermore, the interesting coordination-accelerated biodegradation behaviour of the PEG/rFeOx-HMSN nanocatalyst enabled its rapid biodegradation in vivo and fast elimination from the body.

However, the low efficiency of the Fenton reaction in cancer cells is the main issue limiting the use of iron-based NPs for CDT [83]. To improve the therapeutic efficacy of CDT, several methods have been then proposed to solve this problem [84, 85]. To this end, Zhao et al. developed chelating complex ferrous-cysteine–phosphotungstate nanoparticles (FcPWNPs) to enhance the efficiency of CDT by using cysteine and phosphotungstate for accelerating the electron transfer between Fe^2+^ and Fe^3+^ [35]. This nanoplatforms therefore overcame the pH issue owned by acidity-dependent therapies like CDT, proving the efficiency of the developed nanoplatform also in vivo. In fact, 4T1 tumour-xenografted BALB/c mice were treated with the FcPWNPs injected intratumorally (i.t.) and intravenously (i.v.). FcPWNPs significantly inhibited tumour growth by both i.t. or i.v. administration.

Since the Fenton reaction is strictly dependent also on the concentration of H_2_O_2_ in the tumour tissue, another way to overcome the limitations of CDT is to increase the level of H_2_O_2_ within the tumour, which may not be sufficient to generate enough ^•^OH to damage the tumour cells. For this purpose, an alternative strategy consists in generating H_2_O_2_ in situ using glucose oxidase (GOD), as GOD can exploit the abundant glucose present in the tumour to generate H_2_O_2_ and facilitate the generation of ^•^OH. To this end, Huo et al. developed a nanocomposite consisting of GOD and an iron oxide nanoparticles (IONPs) Fe_3_O_4_ NPs [36]. GOD and Fe_3_O_4_ NPs were incorporated into biodegradable silica NPs (GOD-Fe_3_O_4_@DMSNs). In this case, GOD was able to efficiently deplete glucose within the tumour cells to generate a large amount of H_2_O_2_ to enhance the Fenton-like reaction induced by Fe_3_O_4_ NPs, and thus the resulting highly toxic ^•^OH determined in vivo tumour growth suppression. Indeed, the in vivo therapeutic performance of the GOD-Fe3O4@DMSNs, a biodegradable and biocompatible compound, showed a highly desirable tumour suppression effect against both 4T1 breast cancer xenografts at a dosage of 10 mg kg^−1^ and U87 tumour xenografts at the same dosage. The use of this elaborately designed nanocomposite into the tumor tissue has been showed in this work and this is able to trigger the specific sequential reactions within and under specific TME responses to decrease the tumor growth. Furthermore, this gives an alternative strategy for the efficient tumor therapy with largely enhanced tumor specificity, while reducing side effects to normal tissues and organs.

The use of IONPs in anticancer CDT has then encouraged the investigation of iron-free nanozymes with pH-reactive catalytic actions [86]. For instance, Cu-based nanoparticles possessing peroxidase-mimicking actions, such as CuS and CuO, can trigger Fenton-like reactions by the cycling between Cu^+^ and Cu^2+^ [87–92]. Therefore, it is supposed that Cu-based nanozymes will stand out as multifunctional nanoplatforms thanks to the intrinsic Cu chemical features like potentiation of some drugs (e.g., tetraethylthiuram disulfide) and facilitation of ATP depletion in inhibiting cancer cell growth [93, 94].

**Fig. 10:**
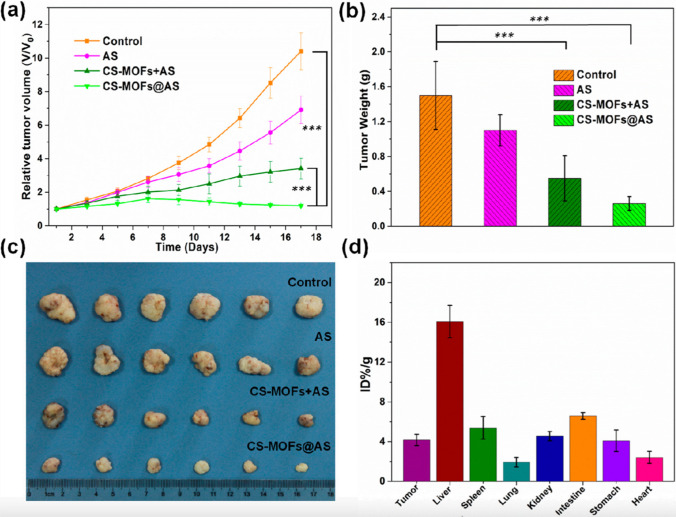
In vivo antitumor effect: **a** tumor growth curves of different groups (PBS, AS alone, CS-MOFs + AS which is a mixture of CS-MOFs and free AS (where AS is nonencapsulated), and CS-MOFs@AS) of tumor-bearing mice after various treatments indicated every 2 days for 17 days, the data are presented as mean ± standard deviation (n = 6); **b** average weights of tumors of each group at the end of different treatment (***p < 0.001, n = 6); **c** photographs of the tumors from different mice groups at day 17 after treatments; and **d** biodistribution of CS-MOFs in HeLa-tumor-bearing mice at 24 h after i.v. injection [37]

Recently, some organic molecules have been proved to possess ROS-generating activities, being responsive to specific biochemical triggers that initiate structural modifications for enabling ROS generation [12]. These bioresponsive organic molecules can innate ROS generation without the H_2_O_2_, therefore overcoming the low amount of H_2_O_2_ in cancer cells, while conventional inorganic Fenton agents necessitate H_2_O_2_ as substrate. Specifically, artemisinin and its derivatives, i.e., artesunate (AS), are the most representative among these organic agents thanks to the reductive cleavage, upon Fe^2+^ activation, of their endoperoxide bridges, resulting in significant ROS production. Thanks to the recent advances in this field, Wang et colleagues designed core–shell Mn3[Co(CN)6]2@MIL-100(Fe) metal–organic frameworks (CS-MOFs) nanocubes, which enabled the synchronous co-delivery of AS and ferric ions in HeLa-tumor-bearing mice [37]. Indeed, CS-MOF nanocubes have been proven to act as both Fe^2+^ donor and AS carrier, resulting in pH-responsive AS activation and intratumoural ROS generation. In this work the nanodrugs at the treatment dose resulted in a much better anticancer efficiency within 17 days (Fig. 10). In vivo results showed that the CS-MOFs@AS tumour efficacy was increased significantly of 5.79 times compared to the free AS exposure. This can suggest that the co-delivery of AS and Fe ions for tumor treatment could be a successful step. All these indications showed that the use of heterogeneous hybridization of two MOFs as drug carriers and Fe-ion suppliers can be considered as a new strategy for tumour treatment.

The accomplishment of artemisinin and its derivatives in CDT has further encouraged the investigation of new kinds of peroxides to trigger ROS production in cancer. One of the main end products of lipid peroxidation is linoleic acid hydroperoxide (LAHP), which can be broken down to ^1^O_2_ in the presence of Fe^2+^ via Russell's mechanism. Indeed, LAHP can be used for anticancer CDT by combining it with Fe-based nanoplatforms [12]. In this regard, Zhou et al. developed a composite LAHP-IONPs (IO-LAHP) nanoplatform for triggering sequential intracellular reactions against cancer. The removal of Fe^2+^ from the IO-LAHP surface is facilitated by the slightly acidic TME, resulting in the cleavage of LAHP into the highly cytotoxic _1_O^2^. The authors showed that the IO-LAHP nanoplatform was able of provoking apoptotic cancer death via the ^1^O_2_ production and the consequent ROS-mediated mechanism, which resulted in U87MG tumor growth inhibition [38]. In conclusion, considering the low amount of endogenous H_2_O_2_ and the subsequent poor ROS production in conventional Fenton-based CDT, the use of LAHP as an exogenous ROS source can significantly increase the intratumoural ROS generation paving the way to the next generation of CDT.

## Technologies for the detection of ROS production in in vivo models

Since the most typically ROS found in biological systems are O_2_^•−^, H_2_O_2_ and ^•^OH, different techniques have been developed to detect them. By considering the different techniques, fluorescence and electrochemical methods are the one that have been extensively developed and used for ROS detection. [95]. The detection of O_2_^•−^ is not directly due to its short half-life and its high reactivity. Indeed, the real-time detection techniques are essential to precisely investigate the impact from a biological point of view of O_2_^•−^ at elevated levels. Several traditional methodologies, like high-performance liquid chromatography (HPLC), electron spin resonance (ESR) and mass spectrometry (MS) are commonly considered for the O_2_^•−^ detection. However, most of these techniques cannot be used for the detection of the in-situ generation of this radical specie, representing therefore a critical disadvantage. The O_2_^•−^ tracking can be performed by fluorescence methodologies, thanks to their high sensitiveness, selectivity and ease feasibility. Yang and colleagues [96] elaborated a NIR fluorescent sensor by observing the emission at 716 nm and a large Stokes shift at 216 nm, which present an increased signal detection compared to the typical emission wavelength (450–550 nm). By considering these wavelengths related to O_2_^•−^ presence, a fluorescent probe was designed according to the luminescence of an aggregation-induced emission (AIE) compound made by dibenzo[a,c]phenazine. In a different work, Huang et al. [97] used a multi-response fluorescent probe for investigating O_2_^•−^ accumulation in biological systems. Thanks to the Hcy-Mito probe, authors got outstanding sensitivity and selectivity for endogenous O_2_^•−^ both in in vitro and in vivo model. This innovative work showed the possibility to locate the presence of O_2_^•−^ with high fluorescent contrast inside mouse bodies, able to reach different profundity of several tissues and organs. Close to the use of fluorescence methods to detect O_2_^•−^, researchers employed alternative solutions to overcome main fluorescence method’s problems like expensiveness and the limited temporal or spatial resolution. Therefore, electrochemical techniques, that use the same principle as the fluorescence method since it combines electrochemical reactions with specific sensing elements for detecting O_2_^•−^ also in its unstable concentrations, have been introduced as an alternative solution [98].

Another reactive specie interesting to be investigated is H_2_O_2_ that shows a longer lifetime, up to minutes, according to the enclosing levels of enzymes that determine the decomposition of H_2_O_2_. H_2_O_2_ can cross cell membranes, reaching cellular compartments by diffusion, and could be even acts like a second messenger in the cell signal transduction by oxidizing the protein thiols, in particular the cysteine thiol in signaling proteins [99, 100]. Even if H_2_O_2_ reacts badly with many biological molecules, since has many kinetic reactions, its harmful outcomes are due to the highly reactive ^•^OH, made in the presence of transition metals, like ferrous ions, via the Fenton reaction. Therefore, H_2_O_2_ itself is not the extremely direct cause of cell damages but ^•^OH from H_2_O_2_ via the Fenton reaction is the main responsible of cytotoxic effects. However, due to its high availability and accessibility, H_2_O_2_ is a commonly used chemical to investigate oxidative stress, becoming a structural part of the current knowledge of this field of study [98]. In the electrochemical techniques, the innovative method used for the detection of H_2_O_2_ aims at modifying the electrode surface with an electrocatalyst that can be either an inorganic compound or an organic biological compound, like hemin, myoglobin or cytochrome-c. Indeed, these compounds contain in their inner core a redox agent heme, that works like an active catalytic element for H_2_O_2_ detection. Therefore, the direct transfer of electrons between the active sites reacting with H_2_O_2_ and the electrode surface is measured as an increased redox response. An alternative approach consists in the immobilization on a large surface area of heme containing biological molecules on highly conductive materials. For example, different combinations between biological elements and metal/metal oxide NPs have been described in several works for a better detection of H_2_O_2_ [101, 102].

Finally, similar to O_2_^•−^, the ^•^OH detection is really challenging because of its high reactivity, extremely short lifetime, and the difficulty to detect this radical specie and to study its relationship to cellular damages. Recently, the fluorescence method showed promising results thanks to high selectivity, sensitivity, and the ability to detect ^•^OH in living cells by a real-time analysis [98]. For example, Feng and colleagues [103] developed a NIR-II fluorescent probe with sensor sensitivity to ^•^OH in in vitro and in vivo models. This probe was characterized by cyanine molecules as sensing element. The reaction between ^•^OH and cyanine molecules is developed at the C-N bonding, inducing fluorescence emission at 1044 nm. A clear imaging of the different level of ^•^OH within mouse organs was obtained by using a limit detection of 0.5 nM. Another example is the one of Yuan and colleagues, that designed and synthesized an innovative mitochondria-targeted fluorescent probe (RThy) to detect ^•^OH in living cells and zebrafish. This probe is based on xanthene derivative with a cation group, which allows to target mitochondria specifically. This probe works by using the specific cleavage reaction by ^•^OH of azo-group [104].

## ROS-generating nanoplatforms towards clinical trial

Prior to clinical use, several points of nanoplatform design need deep investigation and also optimization. Specific pharmacokinetic parameters such as biodistribution and elimination, biocompatibility and residual toxicity need a greater attention compared to what has been know up to nowadays [105]. Other important aspect that needs to be taken into consideration is the huge need to have specific preclinical studies, since normally less than 1% of the NPs administered in a systemic way are able to reach their final target, due to kidney clearance and reticuloendothelial system clearance [106]. Furthermore, tumor architecture possesses an irregular vascular architecture structure that leads to a not homogeneous nanoplatform distribution and therefore treatment. Also, the intrinsic toxicity of materials that are frequently present into these nanoplatforms, to increase the ROS yield, show in vivo toxicities [106]. Although substantial progress has been made in this therapeutic area in recent years to overcome their main limitations and so to close the gap between bench and bedside, with more nanocomposite structures being developed with remarkable results in in vivo animal models, clinical translation remains a critical issue. Indeed, it should be noted that a small number of nanomedicines approved as ROS-based therapies have currently entered clinical trials. The most prominent example is the radio-enhancer NBTXR3 developed from the French company Nanobiotix [107, 108]. NBTXR3 is a hafnium oxide nanoparticle (HaO_2_ NP) with a high atomic number (Z = 72) which make it an efficient radiosensitizer. Because NBTXR3 is physically and chemically inert in biological medium, it can be used safely in biomedical applications. When NBTXR3 reaches tumor tissues and gets RT, its high electron density increases the likelihood that it will interact with the incoming radiation, causing more energy to be deposited inside the irradiated tissues than RT alone and resulting in tumour cell death. Preclinical research has revealed that using NBTXR3 during RT an excellent antitumor activity can be obtained and may enhance patient outcomes in a variety of cancer types [109, 110]. The phase 1 NBTXR3 clinical trial indicated that combining NBTXR3 with RT was a viable therapy strategy [110–112]. In addition, in a multicenter, randomized, controlled phase II/III clinical trial (NCT02379845), the combination of NBTXR3 with RT resulted in excellent radiological and pathologic responses in patients with locally advanced soft tissue sarcoma [111]. However, although other examples can be reported within the therapeutic area covered by this review (e.g., NCT04559685, NCT04845919), as far as we know this is still the only example of nanomedicine approved for ROS-based cytotoxic therapies [113–115]. By considering the translation of ROS-generating nanoplatforms from pre-clinical models to clinical trials, several issues can be further postulated. Many pre-clinical research on ROS-based cancer therapy focuses on creating animal models, which normally have a perfect therapeutic effect in tumor treatment. However, the understanding of the interactions between the ROS-based nanomedicines and the biological environment in vivo are still suffering of a deep and complete knowledge. Furthermore, significant differences are still present in the organizational structures and biological behaviors between experimental animal models and human physiological environments. Crucial is to specifically investigate the efficacy and safety of ROS-based nanoplatforms before moving to the patients [116]. The nanoplatforms used in laboratory and the one that move to clinical stage, are still produced in a small-scale, characterized by drug stability and reproducibility within different batches. Therefore, the shift to a large-scale production involved an increase in testing costs that can burden pharmaceutical companies and researchers [116].

## Summary and outlook

The peculiar properties of ROS have prompted researchers to fully exploit these chemical entities leading to increasingly significant biomedical achievements. Because of the noteworthy progress in nanotechnology, a wide range of nanomaterials with unique ROS-regulating properties have been investigated for biomedical applications to control the ROS spatial–temporal behavior in the biological systems. Nanomedicine has thus promoted the development of a next-generation of advanced therapeutic methodologies based on down- and up-regulation of ROS. In this review, we have summarized, to the best of our knowledge, the most interesting and promising developments in ROS-based cytotoxic therapies exploiting ROS-generating nanoplatforms for cancer treatment. To this end, four different ROS-based cytotoxic therapies have been reviewed: PDT, SDT, RT and CDT. Compared with conventional chemotherapy and radiotherapy, many advantages of various ROS-based cancer therapies, such as high selectivity with potential low toxicity, high penetration depth and non-invasiveness would enable ROS-based therapy to be a more promising therapeutic approach. However, also the disadvantages provided by the stimuli considered in this review need to be considered to understand their limits and how to overcome them (Table 3).

**Table 3:** Summary of the main characteristics of ROS-based cytotoxic therapies for cancer treatment

Therapeutic approach	Stimulus	Advantages	Disadvantages
Photodynamic therapy	Light, NIR	• Localized therapeutics	• Limited penetration capacity
		• Low invasiveness	• Oxygen dependence
Sonodynamic therapy	US	• Increased tumour penetration	• Oxygen dependence
		• Localized therapeutics	• Low US stability of sonosensitizers
		• Reduced side effects	
Radiation therapy	X-ray	• Increased tumour penetration	• Radio-resistance
		• Controllable localization	• Damage to other healthy tissues
			• Oxygen dependence
Chemodynamic therapy	Fenton-based reactions	• High tumor specificity	• Uncontrollable reaction processes
		• Free from tissue penetration limitation	• Limited H_2_O_2_ in tumor tissues
			• Systemic side effects

To address this matter, we believed that several challenges must be face. First the development of more suitable diagnostic techniques for real-time monitoring of ROS content in pathological areas of the human body, in order to establish the appropriate dosages and routes of administration of ROS-generating nanoplatforms. Indeed, for the development of ROS-related nanomedicines, precise tools and methodologies on ROS capable of precisely identifying the ROS dynamic in biological systems are required. Conventional approaches for detecting ROS, i.e., mass spectrometry, high-performance liquid chromatography and electron paramagnetic resonance spectroscopy are not able to provide real-time monitoring, as well as obtaining dynamic information and exact quantification of ROS creation or elimination [117]. Colorimetric, fluorescent, and luminescent probes have been frequently used in the kinetic assessment of ROS reactions in recent years [118]. However, they are non-specific and do not allow to distinguish among different ROS during detection. In particular, the precise detection of ROS in vitro and in vivo remains a significant problem in progressing this scientific area.

Second, even in ROS-based cytotoxic therapies, the nanotoxicity of the nanomaterials used must be investigated and managed. This is mainly because the nanotoxicity of nanomaterials is determined by their physicochemical characteristics (e.g., dimension, surface area, shape, and aggregation) and by their interaction with the environment. Thus, understanding the roles of these factors in determining nanotoxicity could result in indispensable knowledge for the development of safe nanomedicines. Moreover, a systematic analysis of the current strategies for lowering nanotoxicity is essential for the design of safe nanomedicines in this scientific field. Even if the reported nanomaterials show excellent biosafety and biocompatibility in in vivo systems, their chronic toxicities and side effects still need to be systematically and extensively studied to provide therefore long-term biosafety data [119, 120]. This is even more important as the present fashion in nanomedicine design is moving towards the clustering of different therapeutic agents in a single nanoplatform for achieving multifunctionality and high efficiency of ROS regulation (e.g., the creation of sequential catalytic nanoplatform), making more difficult to study the safety of each component.

Third, again, in recent years, the complex structural and functional design of ROS-generating nanoplatforms, will lead to complexity in large-scale preparation that must ensure high repeatability. Indeed, slight variations in the manufacturing process can lead to drastic changes in physicochemical properties, like size, surface, charge, components, even therapeutic results. From the perspective of large-scale preparation, we encourage the development of simple but effective nanomedicines.

Forth, most of the research on nanomedicines that regulate ROS-based cytotoxic therapies focus on how they work. Nevertheless, the understanding of their in vivo behavior is far from being satisfactory. Moreover, the difference between animal models and humans requires the development of innovative approaches for ensuring efficacy and safety prior to clinical investigations [121].

Fifth, the in vivo TME represent a tumour niche characterized by intrinsic factors different form normal tissues, like an increased ROS production, a low pH, a reduced oxygen level and an overexpression of GSH level [122–124]. Furthermore, extremely important is to consider that different types of cancer cells modulate differently their antioxidant capacities, their internal ROS level and finally their redox balance [125], leading therefore to unlike responsiveness to the ROS-generating platform used.

Finally, in recent years numerous publications have witnessed the extraordinary therapeutic potential of various ROS-generating nanoplatforms to induce selective cytotoxicity against cancer cells. Therefore, the selection of the nanoplatforms more suitable for subsequent clinical translation represents a critical issue.

## Conclusions

Research into ROS-based therapies in cancer treatments has been a hot topic over the past few years, thanks to the intensive studies of ROS in cancer prevention and the rapid development of nanotechnologies. Researchers fully use the unique properties of ROS in cancer therapy to contribute to various tumor treatment modalities. Furthermore, considering the previous matters and limitations described, important is to propose few aspects on which focusing further studies for accelerating the translation to the clinical setting of ROS-generating nanoplatforms. First is essential to develop specific intracellular ROS generators and targeting strategies to enhance the therapeutic outcomes, while reducing at the same time the toxic effects. For example, the intracellular organelles targeting like versus the mitochondria or versus the endoplasmic reticulum to induce oxidative stress can be an interesting possibility to understand the relation between reactive species produced and different organelles. Moreover, even if ROS and RNS are currently the most common reactive species produced in cancer therapeutics, the exploitation of novel reactive species needs to be taken into consideration in the design of new therapeutic strategies. Finally, it is important to better clarify the close and complex relationship between tumour architecture, including TME and immune system, and ROS effects.

In conclusion, more feasible diagnostic modalities for monitoring the ROS levels in selected target sites into human body as the simplification of nanostructure construction represent pivotal technological and scientific issues for the successful development of ROS-generating nanoplatforms. This is extremely important for determining the optimal doses of ROS-generating nanomedicines and improving the translational feasibility. In the coming decades, ROS-based cytotoxic therapies have the potential to become front-runner in the fight against cancer.

## References

[1] Ozcan A, Ogun M, Biochemistry of reactive oxygen and nitrogen species. In: Basic principles and clinical significance of oxidative stress. InTech; 2015. 10.5772/61193.

[2] Ferrari CKB, Souto PCS, França EL, Honorio-França AC (2011). Oxidative and nitrosative stress on phagocytes’ function: from effective defense to immunity evasion mechanisms. Arch Immunol Ther Exp (Warsz).

[3] Stone JR, Yang S (2006). Hydrogen peroxide: a signaling messenger. Antioxid Redox Signal.

[4] Gerschman R, Gilbert DL, Nye SW, Dwyer P, Fenn WO (1979). Oxygen poisoning and X-irradiation: a mechanism in common. Science.

[5] Sharifi Rad M, Anil Kumar NV, Zucca P, Varoni EM, Dini L, Panzarini E, Rajkovic J, Tsouh Fokou PV, Azzini E, Peluso I, Prakash Mishra A, Nigam M, El Rayess Y, El Beyrouthy M, Polito L, Iriti M, Martins N, Martorell M, Docea AO, Setzer WN, Calina D, Cho WC, Sharifi Rad J (2020). Lifestyle, oxidative stress, and antioxidants: back and forth in the pathophysiology of chronic diseases. Front Physiol.

[6] de Freitas LF, Nanomaterials for enhanced photodynamic therapy. In: Photodynamic therapy: from basic science to clinical research. IntechOpen; 2021. 10.5772/intechopen.94255.

[7] Liu Y, Shi J (2019). Antioxidative nanomaterials and biomedical applications. Nano Today.

[8] Zhang C, Wang X, Du J, Gu Z, Zhao Y (2021). Reactive oxygen species-regulating strategies based on nanomaterials for disease treatment. Adv Sci.

[9] Lugrin J, Rosenblatt-Velin N, Parapanov R, Liaudet L (2014). The role of oxidative stress during inflammatory processes. Biol Chem.

[10] Bayda S, Adeel M, Tuccinardi T, Cordani M, Rizzolio F (2019). The history of nanoscience and nanotechnology: from chemical-physical applications to nanomedicine. Molecules.

[11] McNeil SE (2005). Nanotechnology for the biologist. J Leukoc Biol.

[12] Yang B, Chen Y, Shi J (2019). Reactive oxygen species (ROS)-based nanomedicine. Chem Rev.

[13] Zhang Y, Qiu N, Zhang Y, Yan H, Ji J, Xi Y, Yang X, Zhao X, Zhai G (2021). Oxygen-carrying nanoparticle-based chemo-sonodynamic therapy for tumor suppression and autoimmunity activation. Biomater Sci.

[14] Phaniendra A, Jestadi DB, Periyasamy L (2015). Free radicals: properties, sources, targets, and their implication in various diseases. Indian J Clin Biochem.

[15] Juan CA, Pérez de la Lastra JM, Plou FJ, Pérez-Lebeña E (2021). The chemistry of reactive oxygen species (ROS) revisited: outlining their role in biological macromolecules (DNA, Lipids and Proteins) and induced pathologies. Int J Mol Sci.

[16] Gorrini C, Harris IS, Mak TW (2013). Modulation of oxidative stress as an anticancer strategy. Nat Rev Drug Discov.

[17] Fang FC (2004). Antimicrobial reactive oxygen and nitrogen species: concepts and controversies. Nat Rev Microbiol.

[18] Trachootham D, Alexandre J, Huang P (2009). Targeting cancer cells by ROS-mediated mechanisms: a radical therapeutic approach?. Nat Rev Drug Discov.

[19] Yanovsky RL, Bartenstein DW, Rogers GS, Isakoff SJ, Chen ST (2019). Photodynamic therapy for solid tumors: a review of the literature. Photodermatol Photoimmunol Photomed.

[20] Canaparo R, Foglietta F, Barbero N, Serpe L (2022). The promising interplay between sonodynamic therapy and nanomedicine. Adv Drug Deliv Rev.

[21] Kim W, Lee S, Seo D, Kim D, Kim K, Kim E, Kang J, Seong KM, Youn H, Youn B (2019). Cellular stress responses in radiotherapy. Cells.

[22] Jia C, Guo Y, Wu F (2022). Chemodynamic therapy via Fenton and Fenton-like nanomaterials: strategies and recent advances. Small.

[23] Hou Z, Zhang Y, Deng K, Chen Y, Li X, Deng X, Cheng Z, Lian H, Li C, Lin J (2015). UV-Emitting upconversion-based TiO_2_ photosensitizing nanoplatform: near-infrared light mediated in vivo photodynamic therapy via mitochondria-involved apoptosis pathway. ACS Nano.

[24] Lv R, Yang D, Yang P, Xu J, He F, Gai S, Li C, Dai Y, Yang G, Lin J (2016). Integration of upconversion nanoparticles and ultrathin black phosphorus for efficient photodynamic theranostics under 808 nm near-infrared light irradiation. Chem Mater.

[25] Zhang H, Zeng X, Li Z (2020). Copper-chalcogenide-based multimodal nanotheranostics. ACS Appl Bio Mater.

[26] Zhang X, Gao H, Wei D, Pei X, Zhang Y, Wang J, Ding D, Chang J, Wu X (2023). ROS Responsive nanoparticles encapsulated with natural medicine remodel autophagy homeostasis in breast cancer. ACS Appl Mater Interfaces.

[27] Zhu P, Chen Y, Shi J (2018). Nanoenzyme-augmented cancer sonodynamic therapy by catalytic Tumor oxygenation. ACS Nano.

[28] Zou W, Hao J, Wu J, Cai X, Hu B, Wang Z, Zheng Y (2021). Biodegradable reduce expenditure bioreactor for augmented sonodynamic therapy via regulating tumor hypoxia and inducing pro-death autophagy. J Nanobiotechnol.

[29] Zhang Z, Liu X, Chen D, Yu J (2022). Radiotherapy combined with immunotherapy: the dawn of cancer treatment. Signal Transduct Target Ther.

[30] Fan W, Bu W, Zhang Z, Shen B, Zhang H, He Q, Ni D, Cui Z, Zhao K, Bu J, Du J, Liu J, Shi J (2015). X-ray radiation-controlled NO-release for on-demand depth-independent hypoxic radiosensitization. Angew Chem Int Ed.

[31] Choi BJ, Jung KO, Graves EE, Pratx G (2018). A gold nanoparticle system for the enhancement of radiotherapy and simultaneous monitoring of reactive-oxygen-species formation. Nanotechnology.

[32] Ding Y, Sun Z, Tong Z, Zhang S, Min J, Xu Q, Zhou L, Mao Z, Xia H, Wang W (2020). Tumor microenvironment-responsive multifunctional peptide coated ultrasmall gold nanoparticles and their application in cancer radiotherapy. Theranostics.

[33] Chen Y, Li N, Wang J, Zhang X, Pan W, Yu L, Tang B (2019). Enhancement of mitochondrial ROS accumulation and radiotherapeutic efficacy using a Gd-doped titania nanosensitizer. Theranostics.

[34] Wang L, Huo M, Chen Y, Shi J (2018). Iron-engineered mesoporous silica nanocatalyst with biodegradable and catalytic framework for tumor-specific therapy. Biomaterials.

[35] Zhao P, Tang Z, Chen X, He Z, He X, Zhang M, Liu Y, Ren D, Zhao K, Bu W (2019). Ferrous-cysteine-phosphotungstate nanoagent with neutral pH fenton reaction activity for enhanced cancer chemodynamic therapy. Mater Horiz.

[36] Huo M, Wang L, Chen Y, Shi J (2017). Tumor-selective catalytic nanomedicine by nanocatalyst delivery. Nat Commun.

[37] Wang D, Zhou J, Chen R, Shi R, Wang C, Lu J, Zhao G, Xia G, Zhou S, Liu Z, Wang H, Guo Z, Chen Q (2017). Core–shell metal–organic frameworks as Fe^2+^ Suppliers for Fe^2+^-mediated cancer therapy under multimodality imaging. Chem Mater.

[38] Zhou Z, Song J, Tian R, Yang Z, Yu G, Lin L, Zhang G, Fan W, Zhang F, Niu G, Nie L, Chen X (2017). Activatable singlet oxygen generation from lipid hydroperoxide nanoparticles for cancer therapy. Angew Chem Int Ed.

[39] Niculescu A-G, Grumezescu AM (2021). Photodynamic therapy—an up-to-date review. Appl Sci.

[40] Correia JH, Rodrigues JA, Pimenta S, Dong T, Yang Z (2021). Photodynamic therapy review: principles, photosensitizers, applications, and future directions. Pharmaceutics.

[41] Chen J, Zhao JX (2012). Upconversion nanomaterials: synthesis mechanism, and applications in sensing. Sensors.

[42] Qiu H, Tan M, Ohulchanskyy T, Lovell J, Chen G (2018). Recent progress in upconversion photodynamic therapy. Nanomaterials.

[43] Wang G, Peng Q, Li Y (2011). Lanthanide-doped nanocrystals: synthesis, optical-magnetic properties, and applications. Acc Chem Res.

[44] Hamblin MR (2018). Upconversion in photodynamic therapy: plumbing the depths. Dalton Trans.

[45] Liu Y, Meng X, Bu W (2019). Upconversion-based photodynamic cancer therapy. Coord Chem Rev.

[46] Hou Z, Deng K, Li C, Deng X, Lian H, Cheng Z, Jin D, Lin J (2016). 808 nm Light-triggered and hyaluronic acid-targeted dual-photosensitizers nanoplatform by fully utilizing Nd3+-sensitized upconversion emission with enhanced anti-tumor efficacy. Biomaterials.

[47] Xu J, Yang P, Sun M, Bi H, Liu B, Yang D, Gai S, He F, Lin J (2017). Highly emissive dye-sensitized upconversion nanostructure for dual-photosensitizer photodynamic therapy and bioimaging. ACS Nano.

[48] Wang T, Zhang H, Liu H, Yuan Q, Ren F, Han Y, Sun Q, Li Z, Gao M (2020). Boosting H_2_O_2_-guided Chemodynamic therapy of cancer by enhancing reaction kinetics through versatile biomimetic Fenton nanocatalysts and the second near-infrared light irradiation. Adv Funct Mater.

[49] Ralls PW, Jeffrey RB, Kane RA, Robbin M (2002). Ultrasonography. Gastroenterol Clin North Am.

[50] Sirsi SR, Borden MA (2014). State-of-the-art materials for ultrasound-triggered drug delivery. Adv Drug Deliv Rev.

[51] Jiang X, Savchenko O, Li Y, Qi S, Yang T, Zhang W, Chen J (2019). A review of low-intensity pulsed ultrasound for therapeutic applications. IEEE Trans Biomed Eng.

[52] Bachu VS, Kedda J, Suk I, Green JJ, Tyler B (2021). High-intensity focused ultrasound: a review of mechanisms and clinical applications. Ann Biomed Eng.

[53] Canaparo R, Foglietta F, Giuntini F, Francovich A, Serpe L (2020). The bright side of sound: perspectives on the biomedical application of sonoluminescence. Photochem Photobiol Sci.

[54] Putterman SJ, Weninger KR (2000). Sonoluminescence: how bubbles turn sound into light. Annu Rev Fluid Mech.

[55] Umemura S, Yumita N, Nishigaki R, Umemura K (1990). Mechanism of cell damage by ultrasound in combination with hematoporphyrin. Jpn J Cancer Res.

[56] Giuntini F, Foglietta F, Marucco AM, Troia A, Dezhkunov NV, Pozzoli A, Durando G, Fenoglio I, Serpe L, Canaparo R (2018). Insight into ultrasound-mediated reactive oxygen species generation by various metal-porphyrin complexes. Free Radic Biol Med.

[57] Sazgarnia A, Shanei A, Eshghi H, Hassanzadeh-Khayyat M, Esmaily H, Shanei MM (2013). Detection of sonoluminescence signals in a gel phantom in the presence of Protoporphyrin IX conjugated to gold nanoparticles. Ultrasonics.

[58] Hompland T, Fjeldbo CS, Lyng H (2021). Tumor hypoxia as a barrier in cancer therapy: why levels matter. Cancers (Basel).

[59] Zhang H, Chen J, Zhu X, Ren Y, Cao F, Zhu L, Hou L, Zhang H, Zhang Z (2018). Ultrasound induced phase-transition and invisible nanobomb for imaging-guided tumor sonodynamic therapy. J Mater Chem B.

[60] Fu J, Li T, Zhu Y, Hao Y (2019). Ultrasound-activated oxygen and ROS generation nanosystem systematically modulates tumor microenvironment and sensitizes sonodynamic therapy for hypoxic solid tumors. Adv Funct Mater.

[61] Yang Y, Fan Z, Zheng K, Shi D, Su G, Ge D, Zhao Q, Fu X, Hou Z (2021). A novel self-targeting theranostic nanoplatform for photoacoustic imaging-monitored and enhanced chemo-sonodynamic therapy. J Mater Chem B.

[62] Feng Q, Li Y, Yang X, Zhang W, Hao Y, Zhang H, Hou L, Zhang Z (2018). Hypoxia-specific therapeutic agents delivery nanotheranostics: a sequential strategy for ultrasound mediated on-demand tritherapies and imaging of cancer. J Control Release.

[63] Zhang C, Xin L, Li J, Cao J, Sun Y, Wang X, Luo J, Zeng Y, Li Q, Zhang Y, Zhang T, Huang P (2022). Metal–organic framework (MOF)-based ultrasound-responsive dual-sonosensitizer nanoplatform for hypoxic cancer therapy. Adv Healthc Mater.

[64] Zou Z, Chang H, Li H, Wang S (2017). Induction of reactive oxygen species: an emerging approach for cancer therapy. Apoptosis.

[65] Tulard A (2003). Persistent oxidative stress after ionizing radiation is involved in inherited radiosensitivity. Free Radic Biol Med.

[66] Kam WW-Y, Banati RB (2013). Effects of ionizing radiation on mitochondria. Free Radic Biol Med.

[67] Kranjc Brezar S, Prevc A, Niksic Zakelj M, Brozic A, Cemazar M, Strojan P, Sersa G (2020). Synergistic effect of cisplatin chemotherapy combined with fractionated radiotherapy regimen in HPV-positive and HPV-negative experimental pharyngeal squamous cell carcinoma. Sci Rep.

[68] Mortezaee K, Narmani A, Salehi M, Bagheri H, Farhood B, Haghi-Aminjan H, Najafi M (2021). Synergic effects of nanoparticles-mediated hyperthermia in radiotherapy/chemotherapy of cancer. Life Sci.

[69] Dar TB, Biteghe FAN, Kakar Bhanot R, Aniogo EC, Malindi Z, Akinrinmade OA, Chalomie NET, Kombe Kombe AJ, Aboughe Angone S, Ndong JMN, Ndong JD (2022). Synergistic effects of radiotherapy and targeted immunotherapy in improving tumor treatment efficacy: a review. Clin Transl Oncol.

[70] Cho B (2018). Intensity-modulated radiation therapy: a review with a physics perspective. Radiat Oncol J.

[71] Grégoire V, Guckenberger M, Haustermans K, Lagendijk JJW, Ménard C, Pötter R, Slotman BJ, Tanderup K, Thorwarth D, van Herk M, Zips D (2020). Image guidance in radiation therapy for better cure of cancer. Mol Oncol.

[72] Hunte SO, Clark CH, Zyuzikov N, Nisbet A (2022). Volumetric modulated arc therapy (VMAT): a review of clinical outcomes—What is the clinical evidence for the most effective implementation?. Br J Radiol.

[73] Sapkaroski D, Osborne C, Knight KA (2015). A review of stereotactic body radiotherapy—Is volumetric modulated arc therapy the answer?. J Med Radiat Sci.

[74] Liu T, Yang K, Liu Z (2020). Recent advances in functional nanomaterials for X-ray triggered cancer therapy. Prog Nat Sci Mater Int.

[75] Zhang X-D, Luo Z, Chen J, Shen X, Song S, Sun Y, Fan S, Fan F, Leong DT, Xie J (2014). Ultrasmall Au_10–12_ (SG)_10–12_ nanomolecules for high tumor specificity and cancer radiotherapy. Adv Mater.

[76] Al-Zaki A, Joh D, Cheng Z, de Barros ALB, Kao G, Dorsey J, Tsourkas A (2014). Gold-loaded polymeric micelles for computed tomography-guided radiation therapy treatment and radiosensitization. ACS Nano.

[77] Dou Y, Guo Y, Li X, Li X, Wang S, Wang L, Lv G, Zhang X, Wang H, Gong X, Chang J (2016). Size-tuning ionization to optimize gold nanoparticles for simultaneous enhanced CT imaging and radiotherapy. ACS Nano.

[78] Chen Y, Yang J, Fu S, Wu J (2020). Gold nanoparticles as radiosensitizers in cancer radiotherapy. Int J Nanomed.

[79] Klein S, Harreiß C, Menter C, Hümmer J, Distel LVR, Meyer K, Hock R, Kryschi C (2018). NOBF_4_-Functionalized Au–Fe_3_O_4_ nanoheterodimers for radiation therapy: synergy effect due to simultaneous reactive oxygen and nitrogen species formation. ACS Appl Mater Interfaces.

[80] Hauser AK, Mitov MI, Daley EF, McGarry RC, Anderson KW, Hilt JZ (2016). Targeted iron oxide nanoparticles for the enhancement of radiation therapy. Biomaterials.

[81] Li Y, Lin T, Luo Y, Liu Q, Xiao W, Guo W, Lac D, Zhang H, Feng C, Wachsmann-Hogiu S, Walton JH, Cherry SR, Rowland DJ, Kukis D, Pan C, Lam KS (2014). A smart and versatile theranostic nanomedicine platform based on nanoporphyrin. Nat Commun.

[82] Fenton HJH (1894). LXXIII—oxidation of tartaric acid in presence of iron. J Chem Soc Trans.

[83] Cao W, Jin M, Yang K, Chen B, Xiong M, Li X, Cao G (2021). Fenton/Fenton-like metal-based nanomaterials combine with oxidase for synergistic tumor therapy. J Nanobiotechnol.

[84] Zhou T, Xu Y, Xing L, Wang Y, Jiang H (2021). A Harmless–Harmful switchable and uninterrupted laccase-instructed killer for activatable chemodynamic therapy. Adv Mater.

[85] Li J, Yi K, Lei Y, Qing Z, Zou Z, Zhang Y, Sun H, Yang R (2020). Al centre-powered graphitic nanozyme with high catalytic efficiency for pH-independent chemodynamic therapy of cancer. Chem Commun.

[86] Bokare AD, Choi W (2014). Review of iron-free Fenton-like systems for activating H_2_O_2_ in advanced oxidation processes. J Hazard Mater.

[87] Gawande MB, Goswami A, Felpin F-X, Asefa T, Huang X, Silva R, Zou X, Zboril R, Varma RS (2016). Cu and Cu-based nanoparticles: synthesis and applications in catalysis. Chem Rev.

[88] Lee H, Lee H-J, Sedlak DL, Lee C (2013). pH-Dependent reactivity of oxidants formed by iron and copper-catalyzed decomposition of hydrogen peroxide. Chemosphere.

[89] Liu Y, Zhen W, Jin L, Zhang S, Sun G, Zhang T, Xu X, Song S, Wang Y, Liu J, Zhang H (2018). All-in-one theranostic nanoagent with enhanced reactive oxygen species generation and modulating tumor microenvironment ability for effective tumor eradication. ACS Nano.

[90] Masarwa M, Cohen H, Meyerstein D, Hickman DL, Bakac A, Espenson JH (1988). Reactions of low-valent transition-metal complexes with hydrogen peroxide. Are they “Fenton-like” or not? 1. The case of Cu+aq and Cr2+aq. J Am Chem Soc.

[91] Pham AN, Xing G, Miller CJ, Waite TD (2013). Fenton-like copper redox chemistry revisited: hydrogen peroxide and superoxide mediation of copper-catalyzed oxidant production. J Catal.

[92] Wang Z, von dem Bussche A, Kabadi PK, Kane AB, Hurt RH (2013). Biological and environmental transformations of copper-based nanomaterials. ACS Nano.

[93] Skrott Z, Mistrik M, Andersen KK, Friis S, Majera D, Gursky J, Ozdian T, Bartkova J, Turi Z, Moudry P, Kraus M, Michalova M, Vaclavkova J, Dzubak P, Vrobel I, Pouckova P, Sedlacek J, Miklovicova A, Kutt A, Li J, Mattova J, Driessen C, Dou QP, Olsen J, Hajduch M, Cvek B, Deshaies RJ, Bartek J (2017). Alcohol-abuse drug disulfiram targets cancer via p97 segregase adaptor NPL4. Nature.

[94] Gui L, Zhou J, Zhou L, Wei S (2018). A smart copper-phthalocyanine framework nanoparticle for enhancing photodynamic therapy in hypoxic conditions by weakening cells through ATP depletion. J Mater Chem B.

[95] Apel K, Hirt H (2004). Reactive oxygen species: metabolism, oxidative stress, and signal transduction. Annu Rev Plant Biol.

[96] Yang J, Liu X, Wang H, Tan H, Xie X, Zhang X, Liu C, Qu X, Hua J (2018). A turn-on near-infrared fluorescence probe with aggregation-induced emission based on dibenzo[a, c ]phenazine for detection of superoxide anions and its application in cell imaging. Analyst.

[97] Huang Y, Yu F, Wang J, Chen L (2016). Near-infrared fluorescence probe for in situ detection of superoxide anion and hydrogen polysulfides in mitochondrial oxidative stress. Anal Chem.

[98] Duanghathaipornsuk S, Farrell EJ, Alba-Rubio AC, Zelenay P, Kim D-S (2021). Detection technologies for reactive oxygen species: fluorescence and electrochemical methods and their applications. Biosensors (Basel).

[99] Lorenzen I, Eble JA, Hanschmann E-M (2021). Thiol switches in membrane proteins: extracellular redox regulation in cell biology. Biol Chem.

[100] Checa J, Aran JM (2020). Reactive oxygen species: drivers of physiological and pathological processes. J Inflamm Res.

[101] Wang Y, Bi C (2014). A novel nitrite biosensor based on direct electron transfer of hemoglobin immobilized on a graphene oxide/Au nanoparticles/multiwalled carbon nanotubes nanocomposite film. RSC Adv.

[102] Radhakrishnan S, Kim SJ (2015). An enzymatic biosensor for hydrogen peroxide based on one-pot preparation of CeO_2_-reduced graphene oxide nanocomposite. RSC Adv.

[103] Feng W, Zhang Y, Li Z, Zhai S, Lv W, Liu Z (2019). Lighting up NIR-II fluorescence in vivo: an activable probe for noninvasive hydroxyl radical imaging. Anal Chem.

[104] Yuan G, Ding H, Sun H, Zhou L, Lin Q (2019). A mitochondrion-targeting turn-on fluorescent probe detection of endogenous hydroxyl radicals in living cells and zebrafish. Sens Actuators B Chem.

[105] Chehelgerdi M, Chehelgerdi M, Allela OQB, Pecho RDC, Jayasankar N, Rao DP, Thamaraikani T, Vasanthan M, Viktor P, Lakshmaiya N, Saadh MJ, Amajd A, Abo-Zaid MA, Castillo-Acobo RY, Ismail AH, Amin AH, Akhavan-Sigari R (2023). Progressing nanotechnology to improve targeted cancer treatment: overcoming hurdles in its clinical implementation. Mol Cancer.

[106] Souris JS, Leoni L, Zhang HJ, Pan A, Tanios E, Tsai H-M, Balyasnikova IV, Bissonnette M, Chen C-T (2023). X-ray activated nanoplatforms for deep tissue photodynamic therapy. Nanomaterials.

[107] Liu W, Chen B, Zheng H, Xing Y, Chen G, Zhou P, Qian L, Min Y (2021). Advances of nanomedicine in radiotherapy. Pharmaceutics.

[108] Song X, Sun Z, Li L, Zhou L, Yuan S (2023). Application of nanomedicine in radiotherapy sensitization. Front Oncol.

[109] Maggiorella L, Barouch G, Devaux C, Pottier A, Deutsch E, Bourhis J, Borghi E, Levy L (2012). Nanoscale radiotherapy with hafnium oxide nanoparticles. Future Oncol.

[110] Bonvalot S, Le Pechoux C, de Baere T, Kantor G, Buy X, Stoeckle E, Terrier P, Sargos P, Coindre JM, Lassau N, Ait Sarkouh R, Dimitriu M, Borghi E, Levy L, Deutsch E, Soria J-C (2017). First-in-Human study testing a new radioenhancer using nanoparticles (NBTXR3) activated by radiation therapy in patients with locally advanced soft tissue sarcomas. Clin Cancer Res.

[111] Bonvalot S, Rutkowski PL, Thariat J, Carrère S, Ducassou A, Sunyach M-P, Agoston P, Hong A, Mervoyer A, Rastrelli M, Moreno V, Li RK, Tiangco B, Herraez AC, Gronchi A, Mangel L, Sy-Ortin T, Hohenberger P, de Baère T, Le Cesne A, Helfre S, Saada-Bouzid E, Borkowska A, Anghel R, Co A, Gebhart M, Kantor G, Montero A, Loong HH, Vergés R, Lapeire L, Dema S, Kacso G, Austen L, Moureau-Zabotto L, Servois V, Wardelmann E, Terrier P, Lazar AJ, Bovée JVMG, Le Péchoux C, Papai Z (2019). NBTXR3, a first-in-class radioenhancer hafnium oxide nanoparticle, plus radiotherapy versus radiotherapy alone in patients with locally advanced soft-tissue sarcoma (Act.In.Sarc): a multicentre, phase 2–3, randomised, controlled trial. Lancet Oncol.

[112] Bagley AF, Ludmir EB, Maitra A, Minsky BD, Li-Smith G, Das P, Koong AC, Holliday EB, Taniguchi CM, Katz MHG, Tamm EP, Wolff RA, Overman MJ, Patel S, Kim MP, Tzeng C-WD, Ikoma N, Bhutani MS, Koay EJ (2022). NBTXR3, a first-in-class radioenhancer for pancreatic ductal adenocarcinoma: report of first patient experience. Clin Transl Radiat Oncol.

[113] Zha B, Yang J, Dang Q, Li P, Shi S, Wu J, Cui H, Huangfu L, Li Y, Yang D, Zheng Y (2023). A phase I clinical trial of sonodynamic therapy combined with temozolomide in the treatment of recurrent glioblastoma. J Neurooncol.

[114] Alsaab HO, Alghamdi MS, Alotaibi AS, Alzhrani R, Alwuthaynani F, Althobaiti YS, Almalki AH, Sau S, Iyer AK (2020). Progress in clinical trials of photodynamic therapy for solid Tumors and the role of nanomedicine. Cancers (Basel).

[115] Anselmo AC, Mitragotri S (2019). Nanoparticles in the clinic: an update. Bioeng Transl Med.

[116] Zhang Q, Luo Q, Liu Z, Sun M, Dong X (2023). Nano-ROS-generating approaches to cancer dynamic therapy: lessons from nanoparticles. Chem Eng J.

[117] Adegoke O, Forbes PBC (2015). Challenges and advances in quantum dot fluorescent probes to detect reactive oxygen and nitrogen species: a review. Anal Chim Acta.

[118] Chen X, Wang F, Hyun JY, Wei T, Qiang J, Ren X, Shin I, Yoon J (2016). Recent progress in the development of fluorescent, luminescent and colorimetric probes for detection of reactive oxygen and nitrogen species. Chem Soc Rev.

[119] Li S, Jiang P, Jiang F, Liu Y (2021). Recent advances in nanomaterial-based nanoplatforms for chemodynamic cancer therapy. Adv Funct Mater.

[120] Oladipo AO, Lebelo SL, Msagati TAM (2023). Nanocarrier design–function relationship: the prodigious role of properties in regulating biocompatibility for drug delivery applications. Chem Biol Interact.

[121] Foglietta F, Canaparo R, Muccioli G, Terreno E, Serpe L (2020). Methodological aspects and pharmacological applications of three-dimensional cancer cell cultures and organoids. Life Sci.

[122] Weinberg F, Ramnath N, Nagrath D (2019). Reactive oxygen species in the Tumor microenvironment: an overview. Cancers (Basel).

[123] Catalano V, Turdo A, Di Franco S, Dieli F, Todaro M, Stassi G (2013). Tumor and its microenvironment: a synergistic interplay. Semin Cancer Biol.

[124] Su Y, Jin G, Zhou H, Yang Z, Wang L, Mei Z, Jin Q, Lv S, Chen X (2023). Development of stimuli responsive polymeric nanomedicines modulating tumor microenvironment for improved cancer therapy. Med Rev.

[125] Sarmiento-Salinas FL, Delgado-Magallón A, Montes-Alvarado JB, Ramírez-Ramírez D, Flores-Alonso JC, Cortés-Hernández P, Reyes-Leyva J, Herrera-Camacho I, Anaya-Ruiz M, Pelayo R, Millán-Pérez-Peña L, Maycotte P (2019). Breast cancer subtypes present a differential production of reactive oxygen species (ROS) and susceptibility to antioxidant treatment. Front Oncol.

